# Treatment response of colorectal cancer liver metastases to neoadjuvant or conversion therapy: a prospective multicentre follow-up study using MRI, diffusion-weighted imaging and ^1^H-MR spectroscopy compared with histology (subgroup in the RAXO trial)

**DOI:** 10.1016/j.esmoop.2021.100208

**Published:** 2021-07-26

**Authors:** A. Uutela, A. Ovissi, A. Hakkarainen, A. Ristimäki, N. Lundbom, R. Kallio, L.M. Soveri, T. Salminen, A. Ålgars, P. Halonen, R. Ristamäki, A. Nordin, R. Blanco Sequeiros, I. Rinta-Kiikka, E. Lantto, J. Virtanen, E. Pääkkö, E. Liukkonen, J. Saunavaara, P. Ryymin, E. Lammentausta, P. Osterlund, H. Isoniemi, RAXO Study Group

**Affiliations:** 1 Department of Transplantation and Liver Surgery, Helsinki University Hospital and University of Helsinki, Helsinki, Finland; 2 Department of Radiology, HUS Medical Imaging Centre, Helsinki University Hospital and University of Helsinki, Helsinki, Finland; 3 Department of Neuroscience and Biomedical Engineering, Aalto University School of Science, Helsinki, Finland; 4 Department of Pathology, HUS Diagnostic Centre and Applied Tumour Genomics, Research Programs Unit, Helsinki University Hospital and University of Helsinki, Helsinki, Finland; 5 Department of Oncology, Oulu University Hospital, Oulu, Finland; 6 Joint Municipal Authority for Health Care and Social Services in Keski-Uusimaa, Home Care Geriatric Clinic and Palliative Care, Hyvinkää, Finland; 7 Department of Oncology, Tampere University Hospital and University of Tampere, Tampere, Finland; 8 Department of Oncology, Turku University Hospital and University of Turku, Turku, Finland; 9 Department of Oncology, Helsinki University Hospital Comprehensive Cancer Center and University of Helsinki, Helsinki, Finland; 10 Department of Radiology, Turku University Hospital and University of Turku, Turku, Finland; 11 Department of Radiology, Oulu University Hospital, Oulu, Finland; 12 Department of Radiology, Medical Imaging Centre Tampere University Hospital and University of Tampere, Tampere, Finland; 13 Department of Medical Physics, Medical Imaging Centre Tampere University Hospital and University of Tampere, Tampere, Finland; 14 Department of Radiology, Päijät-Häme Central Hospital, Lahti, Finland; 15 Department of Pathology/Oncology, Karolinska Institutet and Karolinska sjukhuset - Tema Cancer, Stockholm, Sweden

**Keywords:** colorectal cancer, liver metastasis, neoadjuvant chemotherapy, liver surgery, diffusion-weighted imaging, magnetic resonance spectroscopy

## Abstract

**Background:**

Colorectal cancer liver metastases respond to chemotherapy and targeted agents not only by shrinking, but also by morphologic and metabolic changes. The aim of this study was to evaluate the value of advanced magnetic resonance imaging (MRI) methods in predicting treatment response and survival.

**Patients and methods:**

We investigated contrast-enhanced MRI, apparent diffusion coefficient (ADC) in diffusion-weighted imaging and ^1^H-magnetic resonance spectroscopy (^1^H-MRS) in detecting early morphologic and metabolic changes in borderline or resectable liver metastases, as a response to first-line neoadjuvant or conversion therapy in a prospective substudy of the RAXO trial (NCT01531621, EudraCT2011-003158-24). MRI findings were compared with histology of resected liver metastases and Kaplan–Meier estimates of overall survival (OS).

**Results:**

In 2012-2018, 52 patients at four Finnish university hospitals were recruited. Forty-seven patients received neoadjuvant or conversion chemotherapy and 40 liver resections were carried out. Low ADC values (below median) of the representative liver metastases, at baseline and after systemic therapy, were associated with partial response according to RECIST criteria, but not with morphologic MRI changes or histology. Decreasing ADC values following systemic therapy were associated with improved OS compared to unchanged or increasing ADC, both in the liver resected subgroup (5-year OS rate 100% and 34%, respectively, *P* = 0.022) and systemic therapy subgroup (5-year OS rate 62% and 23%, *P* = 0.049). ^1^H-MRS revealed steatohepatosis induced by systemic therapy.

**Conclusions:**

Low ADC values at baseline or during systemic therapy were associated with treatment response by RECIST but not with histology, morphologic or detectable metabolic changes. A decreasing ADC during systemic therapy is associated with improved OS both in all patients receiving systemic therapy and in the resected subgroup.

## Highlights

• Colorectal liver metastases respond to systemic therapy by shrinking but also by morphological and metabolic changes.
• Advanced MRI technologies (DWI and ^1^H-MRS) might provide more detailed insight into cancer treatment-induced changes.
• We compare changes seen in MRI with histology.
• Reduction of ADC during chemotherapy was associated with improved survival after liver resection.

## Introduction

Liver is the most common metastatic location in metastatic colorectal cancer (mCRC), seen in 68%-71% when metastatic disease is diagnosed.^1^^,^^2^ Without metastasectomy, the prognosis of mCRC is poor. The reported 5-year overall survival (OS) rate after resection ranges between 32% and 50% in population-based studies.^1^^,^^3^ Liver metastases can initially be considered resectable in up to 20% of patients,^4^ and a further 22% may be converted resectable with effective systemic therapy.^5^ Systemic therapy is based on fluoropyrimidines combined with oxaliplatin and/or irinotecan. The addition of targeted agents further improves response rates and thus conversion rates. Vascular endothelial growth factor (VEGF) antibodies, e.g. bevacizumab, or epidermal growth factor receptor (EGFR) antibodies, e.g. cetuximab or panitumumab, are commonly used.^6^, ^7^, ^8^

The treatment of mCRC is multidisciplinary and a key element is detailed anatomic imaging to determine metastatic spread and resectability.^9^ Contrast-enhanced (CE) computed tomography (CT) and magnetic resonance imaging (MRI) are the most widely used imaging tools for preoperative planning in liver surgery. The high soft tissue contrast makes MRI a valuable tool in the characterisation and response evaluation of liver lesions, especially for lesions smaller than 10 mm and even more so in the presence of hepatic steatosis.^10^ Modern MRI techniques combine anatomic and metabolic information of metastases and liver parenchyma. Diffusion-weighted imaging (DWI) describes the microscopic movement of water molecules, which is more limited in tissues with high cell density, intact cell membranes and viscous fluid.^11^^,^^12^ This limited movement leads to high signal on high b-value DWI and low diffusion values on apparent diffusion coefficient (ADC) maps. The ADC correlates inversely to cell density. Changes in tumour cellularity can be detected using DWI and ADC values, and this could be a tool in clinical decision making.^13^

RECIST 1.1 criteria, the gold standard of treatment response evaluation, are based on tumour shrinkage.^14^^,^^15^ The VEGF antibodies cause tumour necrosis by disrupting the pathologic vascular network of the tumour. This will eventually lead to reduction in tumour diameter, but could be detected earlier and more accurately by changes in tumour appearance and metabolic activity.^16^, ^17^, ^18^, ^19^ Morphologic response criteria for CT imaging have been proposed to detect these responses,^16^ but to our knowledge, no corresponding criteria have been applied for MRI.

Hepatic steatosis can to some extent be evaluated with routine preoperative CT and MRI sequences, but a more detailed tissue characterisation needs special techniques.^20^ ^1^H-magnetic resonance spectroscopy (^1^H-MRS) is a non-invasive technique for analysing tissue metabolism and chemical composition, where results are often expressed using choline ratio to other metabolites. A ^1^H-MRS examination can readily be combined with a normal diagnostic liver MRI.

In this prospective study, gadolinium CE-MRI, DWI and ^1^H-MRS were used to assess treatment response of liver metastases, and compared with RECIST response, morphologic MRI response criteria, histopathological findings and survival. A secondary aim was to assess ^1^H-MRS in evaluation of liver steatosis and chemotherapy-induced liver injury.

## Patients and methods

### Study design

The present study is a pre-planned substudy in the prospective nationwide investigator-initiated RAXO study in Finland (NCT01531621, EudraCT2011-003158-24).^21^^,^^22^ In this substudy, inclusion was between December 2012 and September 2018. The protocol (Supplementary Material, available at https://doi.org/10.1016/j.esmoop.2021.100208) was approved by the Ethics Committee at Helsinki University Hospital. The study was conducted in accordance with the Good Clinical Practice and Declaration of Helsinki and monitored independently.

### Patients

The patients were recruited at 4 university hospitals, out of 21 hospitals participating in the study. The inclusion criteria were histologically confirmed CRC with liver-limited metastases, scheduled for first-line oncologic treatment and age over 18 years. A thoraco-abdomino-pelvic CT was used to detect the metastases before inclusion. All patients provided written informed consent. The oncologist, radiologist and physicist identified patients before first appointment at oncology, as MRI was to be carried out after consent to substudy at first visit and before treatment initiation. The protocol included a baseline MRI, which was repeated after 8-12 weeks of systemic therapy. The study flow chart is shown in Supplementary Figure S1, available at https://doi.org/10.1016/j.esmoop.2021.100208. The data cut-off date for follow-up was 4 May 2020.

### Liver MRI protocol

MR examinations were carried out with three Tesla Siemens Verio or Skyra (Siemens Healthineers, Erlangen, Germany) imagers. At least T2-/T1-weighted, in-phase/out-phase, DWI sequences and ^1^H-MRS of the largest or most appropriate metastasis, and in normal-appearing liver, and dynamic images with gadolinium contrast agent CE-MRI were acquired. All the contrast-free sequences were done before injecting the contrast agent. As the heart pulsation may cause artefact especially in the ^1^H-MRS of the left liver lobe, a suitable patient should have an at least 2-cm metastasis in the right liver lobe. In DWI acquisition, a stack of transaxial images, with a slice thickness of 5-6 mm, covering the whole liver was collected applying single-shot echo-planar imaging DWI incorporated with three orthogonal diffusion gradient directions sequentially using three different b-values of 100, 400 and 800 s/mm^2^. The DWI series was obtained before ^1^H-MRS and was utilised in voxel placement for the subsequent ^1^H-MRS. Firstly, a cubic 2 × 2 × 2-cm^3^ MRS voxel was placed in normal-appearing liver parenchyma avoiding lesions and large vascular structures. Secondly, a lesion spectrum was placed in the active tumour area of the largest metastasis avoiding areas appearing necrotic typically in the centre of the large metastases. Point-resolved spectroscopy technique was used for spatial localisation to obtain spectra with an echo time of 30 ms and repetition times of >4000 ms and >8000 ms, for liver parenchyma and lesion spectra, respectively. Two and sixteen signals were averaged for unsuppressed and water-suppressed spectra, respectively. The total duration of this MRI protocol was about 1 h, which is somewhat longer than the regular protocol of 40 min. A navigator belt was used to trigger ^1^H-MRS and DWI acquisitions to the end of exhalation. The exact sequences are listed in Supplementary Table S1, available at https://doi.org/10.1016/j.esmoop.2021.100208.

### DWI and ^1^H-MRS data evaluation

**Figure 1:**
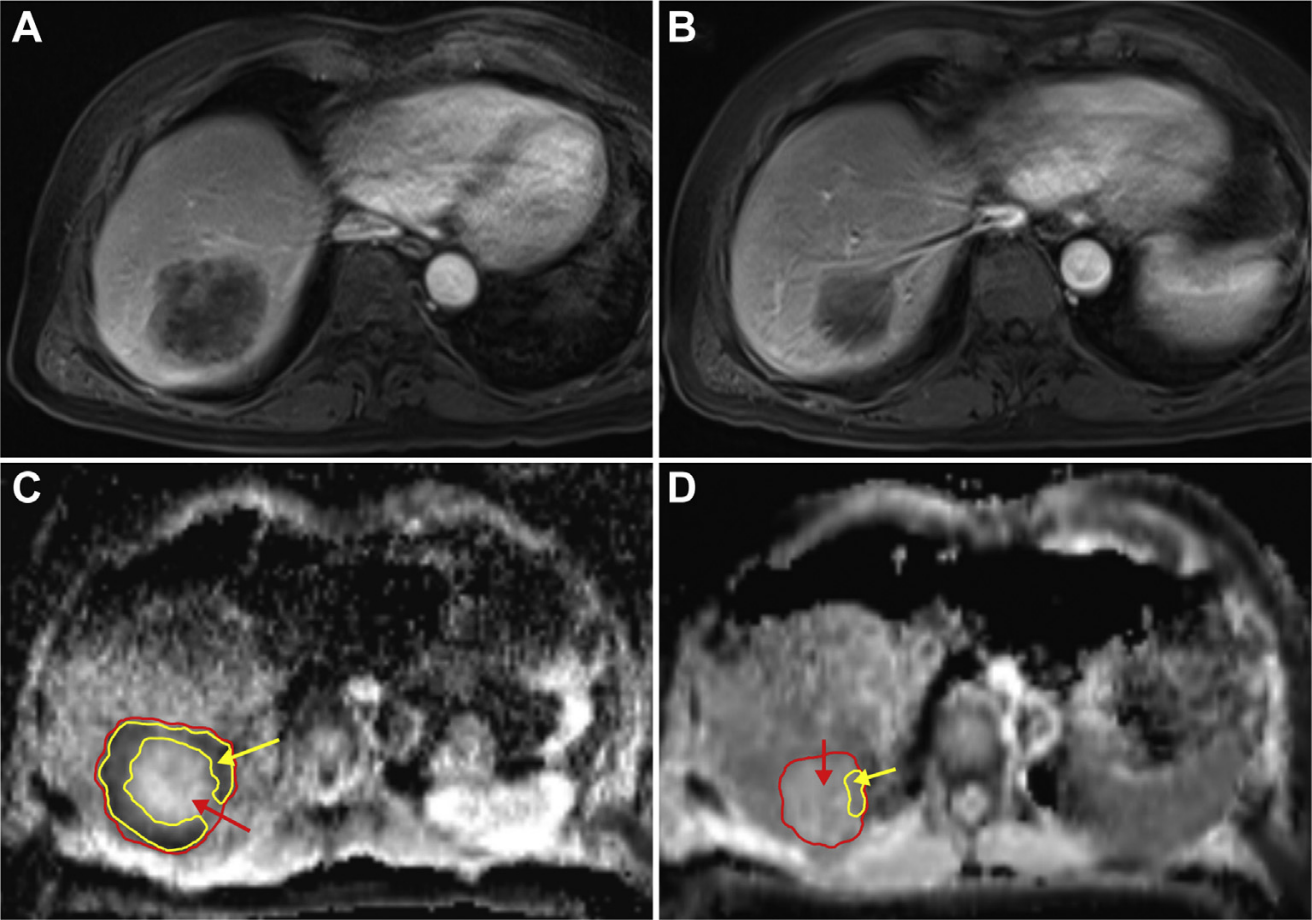
Contrast-enhanced magnetic resonance imaging (MRI) and diffusion-weighted imaging. A gadolinium-enhanced T1 volumetric interpolated breath-hold examination (VIBE) MRI of a liver metastasis before (A) and after (B) chemotherapy. The corresponding apparent diffusion coefficient (ADC) maps calculated from diffusion-weighted images (DWI) of the same tumour before (C) and after systemic therapy (D). Red area and arrow: whole tumour area. Yellow area and arrow: the postulated active tumour area (ADC periphery, the C-formed rim in image C).

DWI and ^1^H-MRS data were analysed independently of the clinical data by a physicist specialised in MRI physics blinded to other clinical data (AIH). The ADC maps were calculated from DWI by using the largest area in the axial plane of the largest liver metastasis and drawing the boundaries of the region of interest (ROI) within the metastasis. The ADC maps were calculated for both the whole metastasis area and for the peripheral part with lowest ADC values. This peripheral lowest ADC part is assumed to present the most cellular area of the tumour. The mean ADC value for ROI (ADC) was used. The measurements were carried out for the same metastasis both before and after systemic therapy (Figure 1). ^1^H-MR spectra were analysed with LCModel v6.3 software (http://lcmodel.ca/lcmodel.shtml) to assess the amount of free choline-containing compounds (CCCs) in the metastasis and fat accumulation in the liver parenchyma outside the tumour.^23^^,^^24^ For assessment of hepatocellular lipids, intensities of methylene and water resonances were determined, and liver fat content was calculated as described previously.^25^ Concentration of CCC was determined from water-suppressed spectra using an unsuppressed water signal as a concentration reference in the LCModel. ^1^H-MRS measurements were done before and after treatment (Supplementary Figure S1, available at https://doi.org/10.1016/j.esmoop.2021.100208).

### CE-MRI evaluation

The MRI was initially evaluated for clinical use by the local radiologists at the university hospitals. A radiologist specialised in liver imaging (AO) re-evaluated the MRIs with RECIST 1.1 criteria and measured the change of largest axial plane area and diameter, and as an experimental endpoint with morphologic criteria adapted to MRI from CT criteria described by Chun et al.^16^ In short, the MRI morphologic response was evaluated using a semi-quantitative three-step scale. A metastasis with heterogeneous attenuation and a thick, poorly defined tumour–liver interface or a peripheral rim of hyperattenuating contrast enhancement was placed in the group 3. A metastasis with homogeneous attenuation with a thin, sharply defined tumour–liver interface or resolution of the enhancing tumour rim belonged to the group 1. The metastases between these two formed the group 2. A change from group 2 or 3 to 1 was considered as optimal response and from 3 to 2 as partial response. This evaluation was done independently from clinical data.

### Histological evaluation

**Figure 2:**
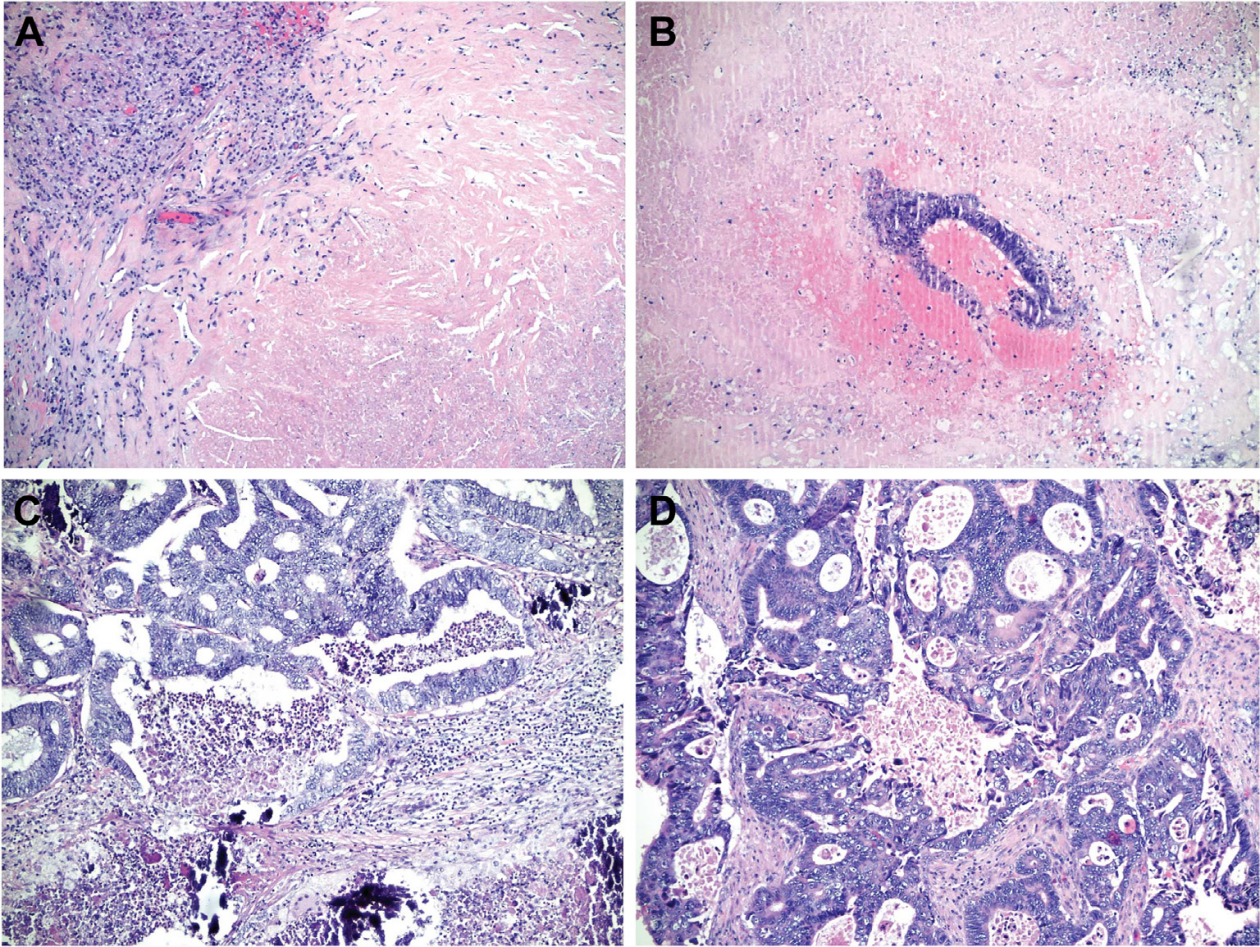
Tumour vitality (TV), tumour regression grade (TRG) and modified tumour regression grade (mTRG) after neoadjuvant or conversion therapy. (A) No vital tumour cells, fibrosis and infarct-like necrosis, TV 0%, TRG1, mTRG1. (B) A small area of vital tumour cells surrounded by large areas of relatively clean (infarct-like) necrosis, TV 10%, TRG2, mTRG2. (C) Larger areas of vital tumour cells bordered by vital stroma and areas of ‘dirty’ (usual) necrosis, with infarct-like necrosis elsewhere in the slide (not shown), TV 30%, TRG3, mTRG3. (D) Mostly vital tumour, with little fibrosis and necrosis, TV 70%, TRG4, mTRG4.

A gastrointestinal pathologist (AR) re-evaluated the histological slides from liver resection specimens independently from radiological and clinical data. A standardised liver resection pathology form was used (Supplementary Table S2, available at https://doi.org/10.1016/j.esmoop.2021.100208). Haematoxylin–eosin, Herovici and periodic acid–Schiff stains were used. Tumour diameter was measured from the original resection block. Tumour vitality was defined as the ratio of vital tumour cells to whole tumour and presented as percent. The tumour histology was further evaluated using tumour regression grade (TRG) and further histological response categories described by Rubbia-Brandt et al.^26^ and by defining distinct types of necrosis: usual necrosis associated with uncontrolled tumour growth and infarct-like necrosis associated with response to chemotherapy (Figure 2).^27^^,^^28^ A modified tumour regression grade (mTRG) was formed by combining TRG with necrosis data.^27^

### Chemotherapy regimens

Standard local treatment protocols based on European Society for Medical Oncology^6^ and National Comprehensive Cancer Network^7^^,^^8^ guidelines were used in the neoadjuvant or conversion chemotherapy (NAC), until disease progression, toxicity or resectability was achieved. In the neoadjuvant setting, oxaliplatin and fluoropyrimidine-based treatment was used,^29^ and in the conversion setting, the most intensive tolerable regimen was used, mostly a doublet or triplet chemotherapy combined with a targeted agent (bevacizumab, cetuximab or panitumumab) based on *RAS* and *BRAF* status.^6^ Bevacizumab treatment was stopped 5-6 weeks before liver resection and one further cycle of chemotherapy was given, as appropriate.

### Statistical analysis

Statistical analyses were carried out using IBM SPSS Statistics version 25 software (IBM Corp., Armonk, NY). Continuous variables with non-normal distribution are presented as median with range, independent samples compared using Mann–Whitney *U* test and related samples using Wilcoxon signed rank test. Categorical variables are presented as absolute numbers (percentage) and compared using chi-square or Fisher's exact test, as appropriate. Risk ratios (RRs) and 95% confidence intervals (95% CI) were calculated to identify the association between ADC (e.g. ADC < 1.20 versus ADC ≥ 1.20) and response variables, and between chemotherapy (NAC + targeted versus NAC no targeted) and response variables. Due to small sample size, the exact methods were applied for CI and for testing the RRs. For response variables with zero cell counts, the zero-count adjustment was done by adding 0.50 to zero cell counts. The RR analyses were carried out using NCSS Statistical Software (2019), LLC (Kaysville, UT). Correlation analysis was compared using Spearman's rho. Follow-up time was calculated using reverse Kaplan–Meier curves. Survival was estimated using Kaplan–Meier curves and analysed by log-rank and Cox regression methods. OS was calculated from the date of diagnosis of mCRC to the date of death from any cause, or censored at last follow-up. Progression-free survival (PFS) was calculated from mCRC diagnosis to relapse after resection/local ablative therapy, progression, death from any cause or censored at follow-up. Two-tailed *P* values <0.05 were considered statistically significant. Receiver operating characteristic analyses were carried out to determine whether mean would be a suitable cut-off for ADC (Supplementary Table S3, available at https://doi.org/10.1016/j.esmoop.2021.100208).

## Results

Fifty-two eligible patients had at least one MRI with DWI and ^1^H-MRS (flow chart in Supplementary Figure S1, available at https://doi.org/10.1016/j.esmoop.2021.100208). Thirty-nine patients had MRI both at baseline and first response evaluation after 2-3 months of first-line therapy. Reverse median Kaplan–Meier follow-up was 65 (95% CI 34-95) months. Demographics are presented in Table 1. Forty patients (77%) had liver resection, of which 37 with complete resection, and 37 had received NAC. Forty-seven received NAC, of which 12 could not be converted and received systemic therapy alone. Partial response on NAC according to RECIST was seen in 51% and stable disease in 49% (Table 2). An optimal morphologic MRI response of the largest lesion was noted in 32%, partial response in 26% and no response in 42%, with corresponding 5-year OS rates of 46%, 51% and 22%, respectively (*P* = 0.092). Median time interval between MRI assessments was 65 days (range 37-174 days) and between the last MRI and operation was 44 days (range 2-234 days).

**Table 1:** Baseline demographics

	All *n* (%)	Liver resection *n* (%)	Systemic therapy only *n* (%)
	52 (100)	40 (77)	12 (23)
Median age (range), years	64.5 (41-80)	62.5 (41-80)	68 (56-77)
Female sex	22 (42)	18 (45)	4 (33)
Male sex	30 (58)	22 (55)	8 (67)
ECOG PS 0	16 (3)	15 (38)	1 (8)
ECOG PS 1	31 (60)	24 (60)	7 (58)
ECOG PS 2	5 (10)	1 (3)	4 (33)
Charlson Comorbidity Index, median (range)	8 (6-13)	8 (6-13)	9 (8-10)
Synchronous metastases	33 (63)	26 (65)	7 (58)
Early metachronous metastases^a^	7 (13)	4 (10)	3 (25)
Late metachronous metastases^a^	12 (23)	10 (25)	2 (17)
Primary in right colon	12 (23)	9 (23)	3 (25)
Primary in left colon	22 (42)	20 (50)	2 (17)
Primary in rectum	18 (35)	11 (28)	7 (58)
Node positive primary	39 (75)	29 (73)	10 (83)
Grade 1	7 (1)	6 (15)	1 (8)
Grade 2	36 (69)	30 (75)	6 (50)
Grade 3	3 (6)	2 (5)	1 (8)
Grade unknown	6 (12)	2 (5)	4 (33)
*KRAS* mutation	17 (33)	14 (35)	3 (25)
*NRAS* mutation of tested^b^	1 (2)	1/32 (3)	0/9 (0)
*BRAF* mutation of tested^b^	7 (13)	3/32 (9)	4/11 (36)
One liver metastasis	20 (38)	16 (40)	4 (33)
Two liver metastases	14 (27)	12 (30)	2 (17)
Three or more liver metastases	18 (35)	12 (30)	6 (50)
Chemotherapy treatment	47 (94)	35 (88)	12 (100)
VEGF antibodies	21 (40)	18 (45)	3 (25)
EGFR antibodies	7 (14)	5 (13)	2 (17)
Time from last MRI to operation in days, median (range)		43 (2-232)	
Elevated CEA at MRI1	36/51 (71)	25/39 (64)	11/12 (92)
Elevated CEA at MRI2	12/38 (32)	5/27 (19)	7/11 (64)
Elevated CEA at operation		8/39 (21)	
Elevated CA19-9 at MRI1	18/38 (47)	14/31 (45)	4/7 (57)
Elevated CA19-9 at MRI2	14/27 (52)	9/20 (45)	5/7 (71)
Elevated CA19-9 at operation		6/20 (30)	

CA19-9, carbohydrate antigen 19-9; CEA, carcinoembryonic antigen; ECOG PS, Eastern Cooperative Oncology Group performance status; EGFR, endothelial growth factor receptor; VEGF, vascular endothelial growth factor.

a Early metachronous from diagnosis to 12 months and late metachronous >12 months from colorectal cancer diagnosis.

b *NRAS* mutation analysed in 41/52, *BRAF* mutation analysed in 43/52.

**Table 2:** Association between ADC at baseline, at response evaluation and change in ADC with NAC versus RECIST and morphologic response criteria, tumour vitality, TRG and mTRG

		All NAC	Baseline ADC			Response ADC			Change ADC with NAC		
			ADC < 1.20	ADC ≥ 1.20	RR (95% CI)^a^	ADC < 1.29	ADC ≥ 1.29	RR (95% CI)^b^	Reduced	Not reduced	RR (95% CI)^c^
Radiology after chemotherapy, *n* (%)		39	13	20		17	18		10	22	
MRI RECIST criteria	Partial response	20 (51)	11 (85)	6 (30)		13 (76)	5 (28)		6 (60)	11 (50)	
	Stable disease	19 (49)	2 (15)	14 (70)	0.22 (0.05-0.65)	4 (24)	13 (72)	0.33 (0.13-0.74)	4 (40)	11 (50)	0.80 (0.33-1.75)
Morphologic MRI criteria^d^	Optimal response	12 (32)	4 (31)	7 (35)		6 (35)	5 (28)		3 (30)	7 (32)	
	Partial response	10 (26)	3 (23)	6 (30)		5 (29)	5 (28)		4 (40)	5 (23)	
	No response	16 (42)	6 (46)	7 (35)	1.32 (0.55-3.04)	6 (35)	8 (44)	0.79 (0.34-1.79)	3 (30)	10 (45)	1.52 (0.53-4.34)
Histology after chemotherapy, *n* (%)		35	13	14		13	11		7	15	
Tumour vitality	0	5 (17)	4 (31)	1 (7)		4 (31)	1 (9)		1 (14)	4 (27)	
	1-30	16 (53)	4 (31)	10 (71)		5 (38)	7 (64)		4 (57)	7 (47)	
	31-100	9 (30)	5 (38)	3 (21)	1.79 (0.57-5.95)^e^	4 (31)	3 (27)	1.13 (0.36-3.77)^e^	2 (29)	4 (27)	1.07 (0.24-3.84)^e^
TRG	1-2	14 (40)	6 (46)	7 (50)		5 (38)	5 (45)		3 (43)	7 (47)	
	3	12 (34)	3 (23)	5 (36)		4 (31)	6 (55)		2 (29)	6 (40)	
	4-5	9 (26)	4 (31)	2 (14)	2.15 (0.55-9.87)^f^	4 (31)	0 (0)	7.08 (0.78-77.6)^f^	2 (29)	2 (13)	2.14 (0.37-12.10)^f^
mTRG	1-2	16 (46)	7 (54)	7 (50)		6 (46)	6 (55)		3 (43)	8 (53)	
	3	12 (34)	3 (23)	6 (43)		4 (31)	5 (45)		3 (43)	5 (33)	
	4-5	7 (20)	3 (23)	1 (7)	3.23 (0.51-38.0)^f^	3 (23)	0 (0)	5.31 (0.65-60.2)^f^	1 (14)	2 (13)	1.07 (0.08-7.17)^f^

ADC, apparent diffusion coefficient; CI, confidence interval; MRI, magnetic resonance imaging; mTRG, modified tumour regression grade; NAC, neoadjuvant or conversion chemotherapy; RR, risk ratio; TRG, tumour regression grade.

a RR = Occurrence of outcome if median ADC <1.20/occurrence of outcome if median ADC ≥1.20.

b RR = Occurrence of outcome if median ADC <1.29/occurrence of outcome if median ADC ≥1.29.

c RR = Occurrence of outcome if ADC reduced with NAC, i.e. decreasing/occurrence of outcome if ADC not reduced, i.e. same or increasing with NAC.

d MRI was not diagnostic for morphology because of motion artefact.

e Comparison groups for RR vitality 0%-30% versus 31%-100%.

f Comparison groups for RR TRG and mTRG 1-3 versus 4-5.

Five patients (13%) had a pathologic complete response, i.e. vitality 0%, TRG1 and mTRG1 (Table 2). A major response to NAC (1%-10% vitality) was seen in 46%, with TG1-2 in 40% and with mTRG1-2 (including necrosis) in 46%.

### Baseline ADC

A baseline ADC measurement of the largest metastasis was available in 40 patients with a median value of 1.20 × 10^−3^ mm^2^/s. A baseline ADC value <1.20 (below median) versus ≥1.20 was associated with partial response on MRI according to RECIST criteria (85% versus 30%; RR 0.22; Table 2). Non-significant inverse trends for associations with baseline ADC values were noted for morphologic responses on MRI, tumour vitality, TRG, mTRG (Table 2) or conversion to resectable. No significant associations with OS or PFS were seen (Supplementary Table S4, available at https://doi.org/10.1016/j.esmoop.2021.100208).

The baseline ADC maps for the peripheral lowest ADC value areas within the metastasis (ADC periphery) had a median of 0.92 × 10^−3^ mm^2^/s, and <0.92 versus ≥0.92 showed no significant associations with RECIST criteria, MRI morphology, metastasis vitality, TRG, mTRG, PFS or OS (Supplementary Tables S4 and S5, available at https://doi.org/10.1016/j.esmoop.2021.100208).

### Response evaluation ADC

After NAC, median ADC at response evaluation was 1.29 × 10^−3^ mm^2^/s in the whole metastasis. ADC <1.29 (under median) versus ≥1.29 was associated significantly with partial response according to RECIST (RR 0.33, Table 2), but was not associated with morphologic MRI criteria or vitality. Response ADC <1.29 showed non-significant inverse trends for association with TRG and mTRG.

After NAC, the median ADC periphery was 1.13 × 10^−3^ mm^2^/s. A reduction of ADC periphery from baseline was associated with partial response (RR 0.40) and showed non-significant inverse trends for TRG and mTRG. No associations with OS or PFS were noted (Supplementary Tables S4 and S5, available at https://doi.org/10.1016/j.esmoop.2021.100208).

### Change in ADC with NAC

A decrease in ADC values (4%-93% decrease from baseline to after NAC) was seen in 10 patients (31%), and showed no association with RECIST, morphologic MRI, tumour vitality, TRG or mTRG (Table 2). TRG and mTRG showed non-significant inverse associations for reduced ADC periphery (Supplementary Table S5, available at https://doi.org/10.1016/j.esmoop.2021.100208). Change in ADC showed no association with fibrosis (*P* = 1.00) or steatosis (*P* = 1.00) in histology.

**Figure 3:**
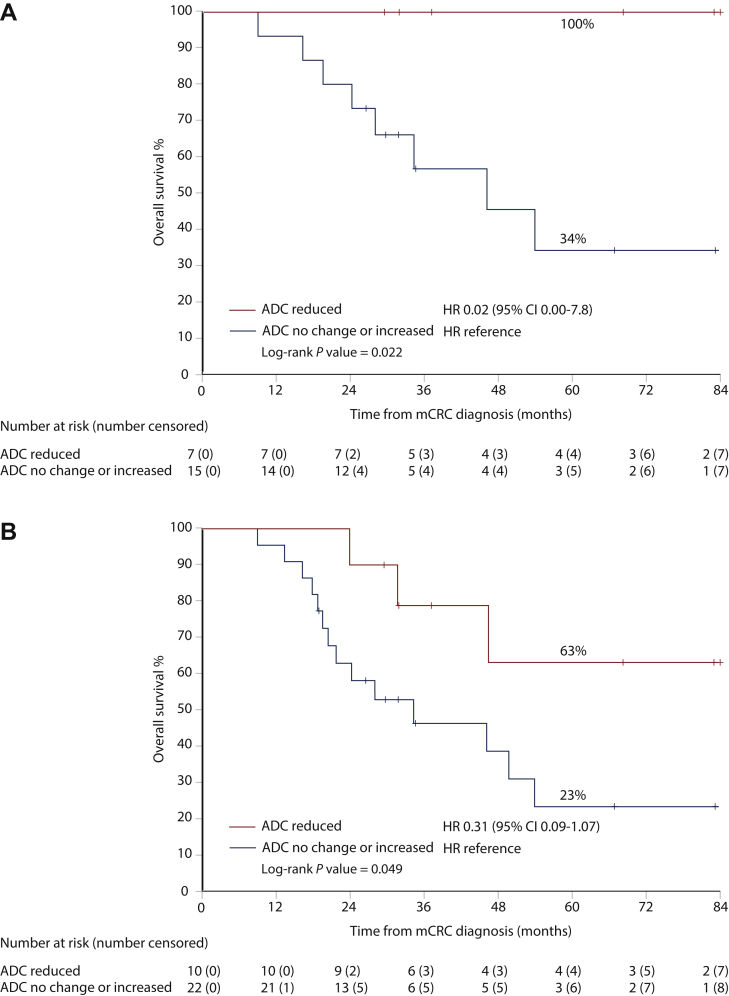
Overall survival in patients whose liver metastases show a reduction versus increase or no change in the ADC after chemotherapy. (A) Resected patients. (B) All patients treated with neoadjuvant or conversion therapy. ADC, apparent diffusion coefficient; CI, confidence interval; HR, hazard ratio; mCRC, metastatic colorectal cancer.

Patients with a decrease in the ADC values had better OS than patients with an increase or no change in ADC (Supplementary Table S4, available at https://doi.org/10.1016/j.esmoop.2021.100208, Figure 3). In patients with liver resection, a decrease in ADC versus increase or no change were associated with 5-year OS rates of 100% and 34%, respectively (*P* = 0.022, Figure 3A). An OS advantage for decreased ADC was seen also in patients receiving NAC with/without resection, with 5-year OS rates of 63% versus 23%, respectively (*P* = 0.049, Figure 3B). An OS advantage for decreased ADC was seen in patients receiving NAC with targeted agents (*n* = 6 decreased ADC versus 13 increased or no change, *P* = 0.029). A decrease in ADC periphery values versus increased or no change did not show a difference in OS (Supplementary Table S4, available at https://doi.org/10.1016/j.esmoop.2021.100208).

Response evaluation ADC under a median of 1.29 was associated with better 3-year PFS (42% versus 17%), but no other significant differences in PFS between groups were noted (Supplementary Table S4, available at https://doi.org/10.1016/j.esmoop.2021.100208).

A decrease in the ADC after NAC showed no statistical difference in DFS after resection (*n* = 6 decreased versus 13 increased or no change), with 5-year DFS rates of 67% versus 45%, respectively (*P* = 0.273).

### NAC with or without targeted agents

Targeted agents with VEGF antibodies (bevacizumab, *n* = 21), EGFR antibodies (cetuximab or panitumumab, *n* = 8), both (*n* = 1) and combination chemotherapy only (*n* = 6) were given. ADC decrease did not show any significant differences for chemotherapy with targeted agents or without. Responders versus non-responders had no significant difference in fibrosis (*P* = 0.47) or steatosis (*P* = 0.57) in histology.

Patients receiving NAC including targeted agents had a higher likelihood of having low tumour vitality 0%-10% (RR 7.17, 95% CI 1.12-66), mTRG1-2 (RR 7.17, 95% CI 1.12-66) and a trend for TRG1-2 (RR 6.28, 95% CI 0.96-58), when compared to patients receiving chemotherapy alone.

### Survival, MRI response and histology

Tumour vitality, TRG or mTRG were not significantly associated with OS or PFS (Supplementary Table S4, available at https://doi.org/10.1016/j.esmoop.2021.100208). Response on MRI according to RECIST or morphologic criteria was not significantly associated with tumour vitality, TRG or mTRG (Supplementary Table S6, available at https://doi.org/10.1016/j.esmoop.2021.100208).

### ^1^H-MRS

A ^1^H-MRS was evaluated in 21 patients, of which 14 were operated after NAC. Although decreases in choline levels in ^1^H-MRS seemed to reflect response to NAC in some patients (Supplementary Figure S2, available at https://doi.org/10.1016/j.esmoop.2021.100208), no significant associations were detected between the baseline relative amounts of free choline versus response on RECIST, morphologic MRI criteria, tumour vitality, TRG or mTRG after NAC. Seven patients had a second ^1^H-MRS after NAC, with a decrease in free choline in four with partial response in RECIST, without statistical significance.

The ^1^H-MRS estimation of liver adiposity, i.e. accumulation of fat in the liver, in the MRI after NAC was associated with macrovesicular steatosis in histological evaluation of the liver resection specimens (*n* = 20, *P* < 0.001, *R*^2^ linear = 0.666 when MRI was done within 60 days before liver surgery, Supplementary Figure S3, available at https://doi.org/10.1016/j.esmoop.2021.100208). According to ^1^H-MRS analysis, liver steatosis increased in 86% of patients during NAC (*n* = 21, *P* = 0.001).

## Discussion

In this prospective study of CRC patients with liver metastases, we analysed systemic treatment-induced morphologic and metabolic changes in CE-MRI, including ADC values of DWI-MRI and ^1^H-MRS, in comparison with histology of resected liver metastases as ‘gold standard’ and in relation to survival.

Patients with a baseline ADC below median had better treatment responses according to RECIST in the present study. This is in line with earlier studies comparing RECIST or similar size-based criteria.^11^^,^^30^, ^31^, ^32^, ^33^ There is no significant association of ADC and morphologic MRI criteria for response, and to our knowledge this has not been studied before. Lower ADC represents higher cellularity and thus possibly a more responding lesion.^11^^,^^12^ Low pre-treatment ADC was not statistically associated with OS or PFS in our study, in line with findings by Tam et al.,^34^ but contrary to the study by Heijmen et al., where low baseline ADC was associated with shorter OS and PFS in patients receiving chemotherapy.^35^ Baseline low ADC is counterintuitive for shorter OS and PFS, as low ADC has consequently shown association with better responses,^11^^,^^30^, ^31^, ^32^, ^33^ a strong positive predictor for better PFS and OS.^14^

Low ADC values after NAC also indicated better treatment response according to RECIST, but not according to morphologic MRI criteria. This is in line with previous findings in rectal primaries treated with chemotherapy,^36^ but there low ADC in responders after 10-12 weeks of chemotherapy was considered loss of non-viable fraction of the treated tumour. Non-responders had high ADC after chemotherapy.^36^ Low ADC could also represent increased fibrosis or steatosis in the tumour tissue due to treatment response at 10-12 weeks, when tumour cells have been replaced with collagen, and thus low motility of water molecules. Low ADC values have been shown in liver cirrhosis, i.e. fibrous tissue with high collagen content.^37^ We noted an improved PFS for patients with low ADC at response evaluation. This inverse correlation between ADC and treatment response and outcome is rarely reported in other studies.

ADC change did not significantly associate with response according to RECIST after a median of 9 weeks of NAC. In early evaluation time point, two studies are in line with our findings (1-2 weeks between imaging),^35^^,^^38^ whereas two studies show that an elevation in ADC during chemotherapy predicts better response (0.5-2 weeks' imaging interval).^30^^,^^39^ Increase in ADC at an early evaluation time point probably measures changes in cellularity and possibly inflammation. Later response evaluation of liver metastases (after 9-15 weeks), as in our study, has been evaluated in one prior small study (*n* = 20).^11^ The findings at baseline are in line, as low baseline ADC correlates with better response, but change was contrary to our findings, as increasing ADC was associated with better treatment response.^11^ The rectal cancer study is contrary to both our and Koh et al.'s studies, as a decreasing ADC is linked to better chemotherapy response.^36^ Low response ADC or decreasing ADC during chemotherapy could theoretically be linked to increasing fibrosis or steatosis in treatment response, but this could not be histologically verified in our small subgroup analyses.

Decreasing ADC associates with better OS in our study, both in resected and in patients receiving systemic therapy with/without targeted agents. Contrary to the present study, two studies report that ADC change was not predictive for OS with 1-week^35^ or 12-week imaging intervals.^34^ In line with these findings, even though the patients with low response ADC had longer PFS, we found no significant difference in PFS whether ADC decreased with systemic therapy or not.

Only a few studies using histology as a reference for ADC behaviour after preoperative systemic therapy are available. Low ADC values are thought to present vital tumour area.^30^ Chiaradia et al.^40^ show that high ADC correlates with tumour necrosis after chemotherapy, but generally not with tumour vitality because of varying amounts of fibrosis and scattered distribution of tumour cells, this is in line with the non-significant trends for TRG and mTRG in our study. Some correlation with ADC and vitality has been noted in patients treated with targeted agents.^40^ Donati et al.^41^ reported that high ADC of the whole tumour or tumour periphery after NAC correlates inversely with low tumour vitality and TRG1-2. Wagner et al.^42^ found no association between whole metastasis ADC and histology after NAC, but ADC of the periphery was higher for metastases with major histological response. A study by Dunet et al.^43^ shows that positron emission tomography findings after NAC correlate with TRG, but do not associate with ADC. These earlier studies comparing ADC with histology reported only MRI findings after NAC and thus the importance of changes in ADC could not be compared. An elevation in ADC value is thought to present a higher degree of freedom of water molecules in the tissue, which could be due to increased necrosis and reduced cellularity.^44^ Our study shows only non-significant inverse trends in histological associations with changes in ADC during NAC versus tumour necrosis, e.g. tumour vitality, TRG or mTRG. Response by RECIST or morphologic criteria did not capture responses in histology, neither with vitality, TRG or mTRG in this population. Histology, on the other hand, did not associate with OS or PFS either. ^1^H-MRS can be used to analyse the amount of free choline and other CCCs and it is usually clearly higher in the liver metastasis than in the surrounding liver parenchyma,^23^^,^^45^ but it has not widely been studied in this setting. In our study, no association was detected between the relative amounts of free choline in the largest metastasis at baseline versus response according to RECIST or morphologic MRI criteria, neither in histological findings. Future studies are needed to evaluate its role in detecting treatment efficacy.

The use of ^1^H-MRS in detecting hepatic steatosis is better described,^24^ and the present study agrees with earlier reports where ^1^H-MRS evaluation of liver adiposity correlates well with histology.^46^ Patients also seem to acquire fat in their livers during NAC, which is a known side-effect of chemotherapy agents.^47^

The strength of this study lies in its prospective multicentre design with repeated assessment, standardised MRI methodology and uniform radiological and histological re-evaluation at a highly specialised tertiary liver centre. Identification of patients was challenging as eligibility was to be checked by oncologist, radiologist and physicist at the time of referral before first appointment at oncology, as postponing treatment due to study procedures was not acceptable. This substudy also needed excellent MRI facilities which were available only in four university hospitals. To our knowledge, this small series is still the largest prospective, multicentre study of repeated DWI with histological correlation, combined with comprehensive survival data of patients treated with chemotherapy and/or resection. The small subgroups due to the prospective nature of the study and technical challenges, especially with repeated DWI and ^1^H-MRS, make the present findings hypothesis generating. A further limitation is that we analysed only the largest lesion and heterogeneity between metastases seen in one-fifth of metastases,^48^ which thus was possibly missed. The ongoing DREAM study (NCT02781935) would, if completed, be the largest study to evaluate MR DWI in detecting chemotherapy response of colorectal liver metastases.

### Conclusions

Liver metastases respond to chemotherapy and targeted agents not only by shrinking in size but also by changes in morphology and metabolic activity, which associates with survival. Conventional imaging modalities might be insufficient in detecting treatment response and MR DWI, especially low or decreasing ADC, provides additional information also for survival, but do not associate significantly with histological findings.

## Supplementary data

Supplementary Material

Supplementary material. Trial registration data
