# Supplementary material for: Treatment response of colorectal cancer liver metastases to neoadjuvant or conversion therapy: a prospective multicentre follow-up study using MRI, diffusion-weighted imaging and 1H-MR spectroscopy compared with histology (subgroup in the RAXO trial)
Source: ESMO Open. 2021 Jul 26;6(4):100208. doi: 10.1016/j.esmoop.2021.100208 (PMC8332656; doi:10.1016/j.esmoop.2021.100208)
Supplement: Supplementary Material [file mmc1.pdf]

Supplementary figure 1. Flow chart for magnetic resonance imaging (MRI) with diffusion weighed imaging (DWI) and ^{1}H-magnetic resonance spectroscopy (^{1}H-MRS, done together with MRI). ADC: Apparent diffusion coefficient. RFA: Radiofrequency ablation. TACE: Transarterial chemoembolization**.**

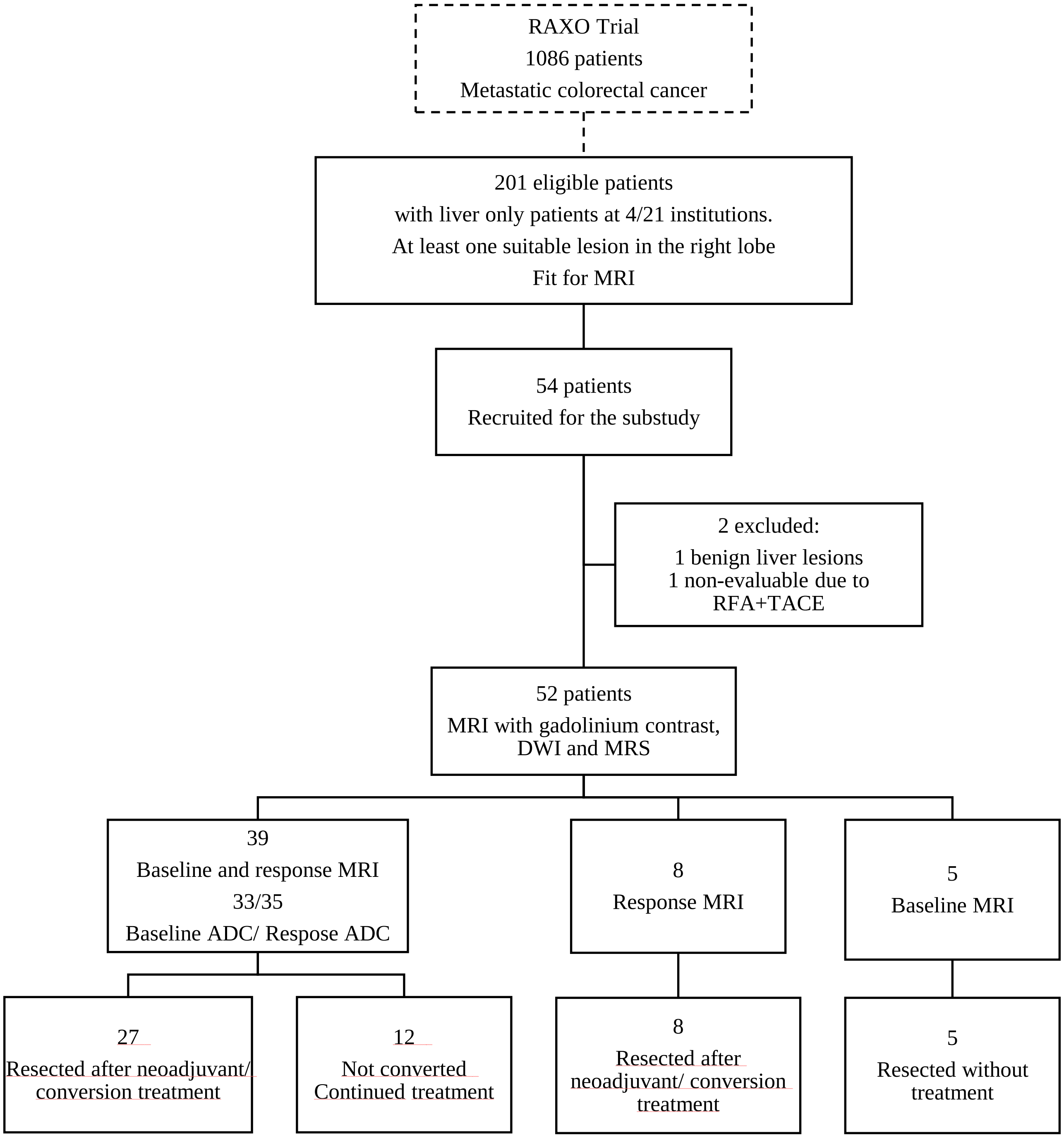

Supplementary figure 2. ^{1}H-MR spectroscopy of a liver metastases in one patient before (left) and after (right) chemotherapy. Upper row: Choline (Cho) resonance decreased by 30%. Lower row: Fat resonance increased to over 4-fold (from 1.4% to 6.9%).

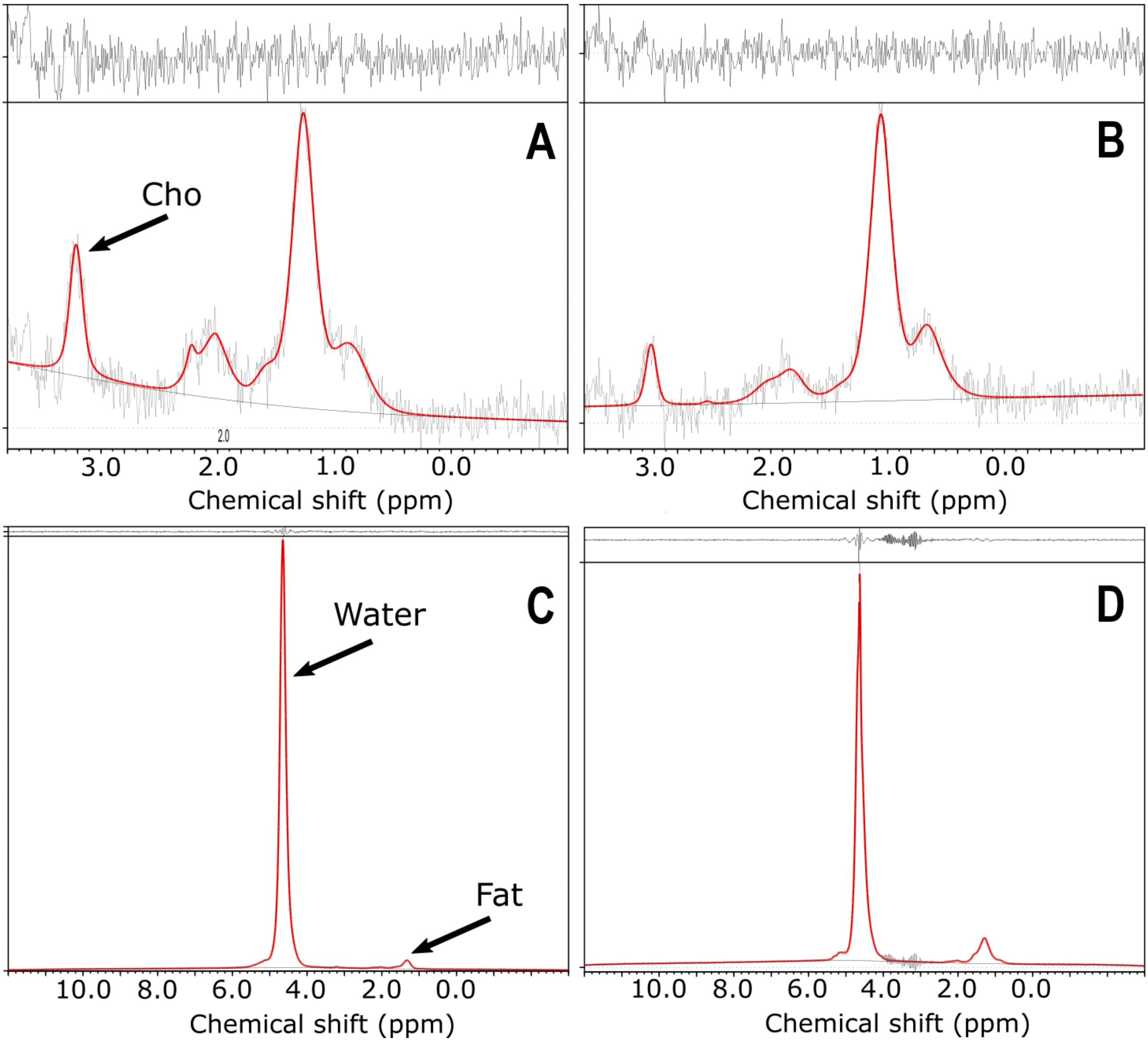

Supplementary figure 3. Scatter plot of the correlation of liver steatosis detected with 1H-MRS and macrovesicular steatosis seen in the actual pathology specimen outside of the tumour area (n=20, p<0.001 Spearman's rho, R2 linear=0.666).

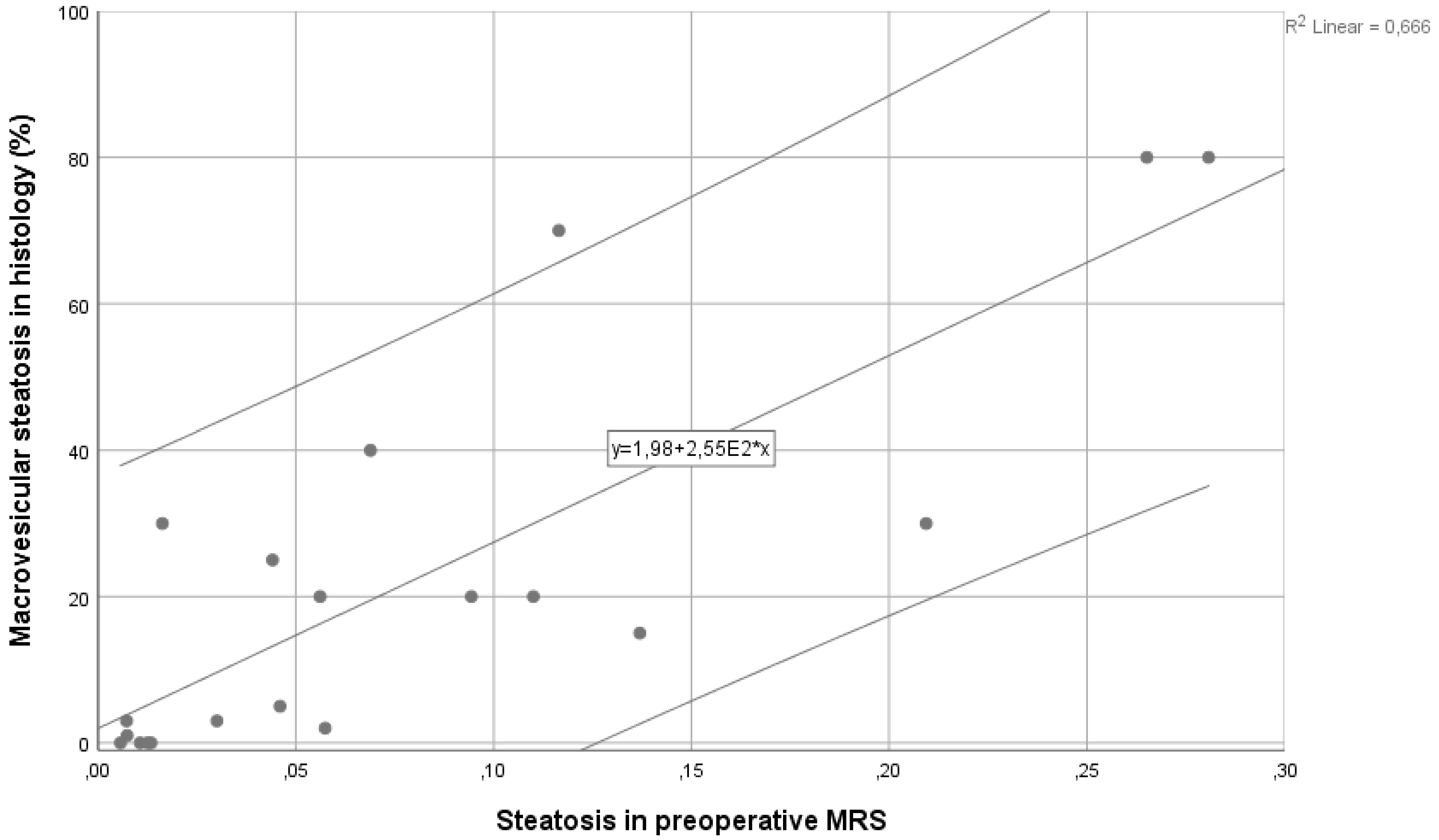

Supplementary table 1. MRI Magnetic resonance imaging (MRI) and ^{1}H magnetic resonance spectroscopy (^{1}H-MRS) sequences used in Helsinki University Hospital.

Sequence name	TR (ms)	TE (ms)	Flip angle (deg.)	Resolution (mm)	Averages	Parallel imaging	Motion control	Fat saturation	DWI b-factors (s/mm2)
Cor T2w Haste	1400	93	160	2,2x1,6x5,0	1	3	Breath hold	no	N/A
Tra T2w Haste	1800	94	160	1,7x1,2x5,0	1	2	Breath hold	no	
Tra T1w Vibe dixon water only	5,47	2,45/3,675	9	1,7x1,2x3,5	1	2	Breath hold	Yes (Dixon)	
Tra T1w Vibe	4,56	2,01	9	1,8x1,3x3,5	1	2	Breath hold	no	
Tra DWI SE EPI	8900	64		3,1x2,5x6,0	4	2	Resp. trigger	Yes (SPAIR)	100, 400, 800
MRS PRESS (svs se)	> 4000	30	90	20x20x20	2		Resp. trigger	no	
MRS PRESS (svs se)	> 4000	30	90	20x20x20	16		Resp. trigger	yes	
MRS PRESS (svs se)	> 8000	30	90	20x20x20	2		Resp. trigger	no	
MRS PRESS (svs se)	> 8000	30	90	20x20x20	16		Resp. trigger	yes	
Tra T1w Vibe	4.56	2,01	9	1,9x1,3x3,5	2	2	Breath hold	Yes (Q-fat sat.)	
Tra T1w Vibe	4.56	2,01	9	1,9x1,3x3,5	2	2	Breath hold	Yes (Q-fat sat.)	
Tra T1w Vibe	4.56	2,01	9	1,9x1,3x3,5	2	2	Breath hold	Yes (Q-fat sat.)	
Tra T1w Vibe	4.56	2,01	9	1,9x1,3x3,5	2	2	Breath hold	Yes (Q-fat sat.)	
Tra T2w Haste	1800	94	160	1,5x1,1x5,0	1	2	Breath hold	Yes (SPAIR)	
Tra T1w Vibe	3,92	1,92	9	1,8x1,3x3,1	1	2	Breath hold	Yes (Q-fat sat.)	

Supplementary table 2. Helsinki University Hospital (HUS) Standard Liver Tumour Pathology Form and Additional Study Pathology Form (sample). Every patients' pathology samples were reanalysed according to HUS Form (in clinical use for HUS patients and Additional Study pathology chart by an experienced gastrointestinal pathologist (AR) and primary investigator (AU).

**STANDARD PATHOLOGY FORM: Liver Tumour pathology chart (sample)**						
**Liver tumour pathology chart**						
**Diagnoses**	Liver, seg. II-III + seg. IVa/VIII partial: metastatic adenocarcinoma					
**Weight of the sample (g)**	235 + 65 g					
**Number of tumours**	2					
**Tumours A….F**	**A**	**B**	**C**	**D**	**E**	**F**
Tumour diameter (mm)	22	15				
Tumour vitality (%)	20	10				
Resection margin (mm)	12	5				
Vascular invasion (+/-)	-	-				
Neural invasion (+/-)	-	+				
Biliary tract invasion (+/-)	-	+				
**Liver parenchyma**						
Inflammation						
Portal (0-3)	1	1				
Interphase (0-3)	0	0				
Lobular (0-3)	0	0				
Fibrosis stage (0-4)	2	2				
**Vacuolization (steatosis)**						
Macrovesicular (%)	5	5				
Microvesicular (%)	0	0				
**Free text**	The tumour consists of glandular cells. Central parts of the tumours are necrotic, vital tumour is seen in the periphery of the tumours. The smaller tumour has several foci of neural invasion and biliary tract invasion is seen.<br>Liver parenchyma outside the tumour has variable portal inflammation and increased fibrosis connecting widened portal areas. Some of the hepatocytes contain adipose vesicles.					
**ADDITIONAL STUDY PATHOLOGY FORM**						
**Largest tumour**	**A**					
Vital tumour cells peripheral/central	peripheral					
Infarct-like necrosis (0-3)	3					
Usual necrosis (0-3)	0					
Tumour fibrosis (0-3)	2					
Modified tumour regression grade	3					
Dangerous halo (+/-)	+					
3 zonal change (+/-)	+					
Mucous	-					
Peritumoral inflammation	-					
**Liver parenchyma**						
Sinus dilatation (+/-)						

Supplementary table 3. Receiver operating characteristics (ROC) analyses for ADC cut-off values.

A. Baseline ADC (n=33)

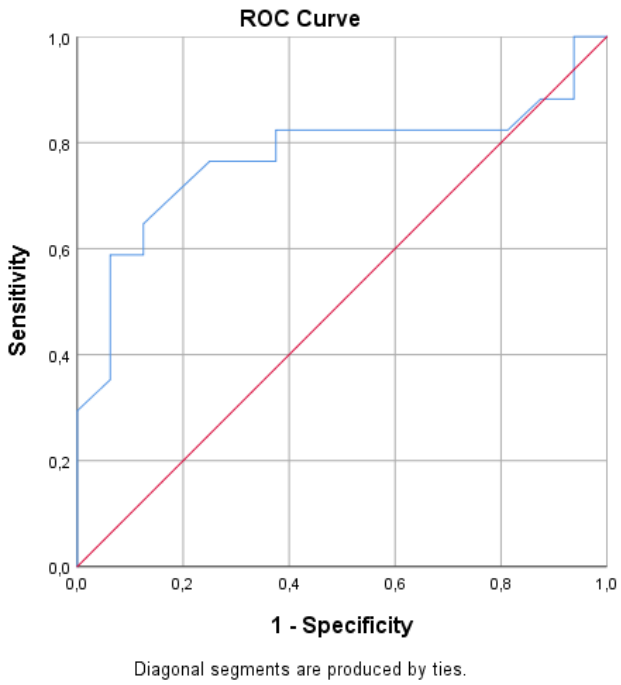

Baseline ADC	Se	Sp	distance to corner
≤1.17	0,65	0,88	0,374
**≤1.20**	**0,71**	**0,81**	**0,349**
**≤1.22**	**0,76**	**0,75**	**0,343**
≤1.27	0,76	0,69	0,391

B. ADC after neoadjuvant or conversion therapy (NAC) (n=35)

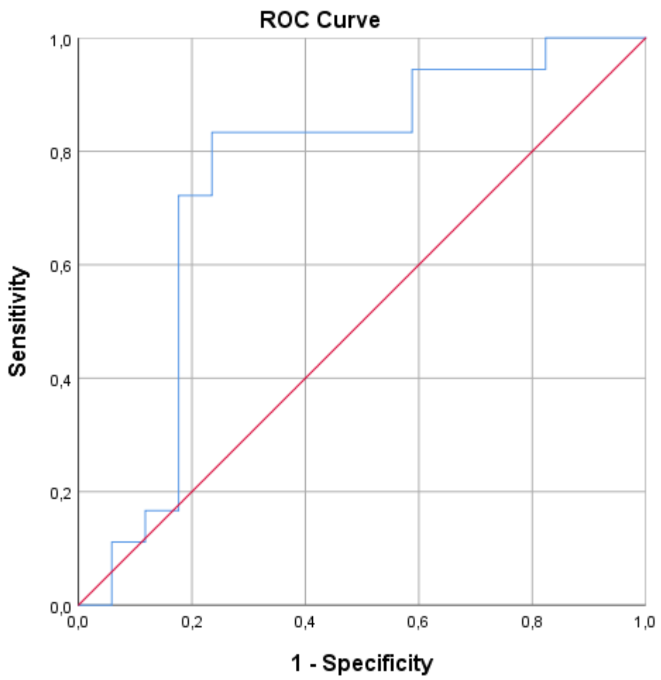

ADC after NAC	Se	Sp	distance to corner
≤1,16	0,67	0,82	0,377
≤1,22	0,72	0,82	0,329
≤1,26	0,72	0,76	0,364
**≤1,29**	**0,78**	**0,76**	**0,324**
**≤1,30**	**0,83**	**0,76**	**0,288**
≤1,31	0,83	0,71	0,338

Supplementary table 4. 5-year overall survival (OS) rate and 3-year progression free survival (PFS) according to ADC at baseline and response evaluation, ADC change, MRI RECIST tumour vitality, TRG and mTRG.

		5-year OS-rate				3-year PFS-rate			
	N	%	vs	%	HR (95% CI)*	%	vs	%	HR (95% CI)*
Resected vs Not resected patients	40 vs 12	55 %	vs	0 %	0.14 (0.06-0.36)	35 %	vs	13	0.47 (0.23-0.96)
Baseline ADC <1.20 vs ≥1.20	21 vs 19	43 %	vs	41 %	0.84 (0.35-2.03)	32 %	vs	24 %	0.61 (0.29-1.23)
Baseline ADC periphery <0.92 vs ≥0.92	15 vs 16	51 %	vs	36 %	0.71 (0.25-1.98)	32 %	vs	25 %	0.76 (0.33-1.74)
Response ADC <1.29 vs ≥1.29	17 vs 18	42 %	vs	26 %	0.67 (0.27-1.68)	42 %	vs	17 %	**0.40 (0.18-0.92)**
Response ADC periphery <1.13 vs ≥1.13	12 vs 12	44 %	vs	33 %	0.95 (0.32-2.84)	16 %	vs	42 %	1.34 (0.50-3.63)
ADC change: Reduced vs Not reduced (all NAC)α	10 vs 22	63 %	vs	23 %	**0.31 (0.09-1.07)**	50 %	vs	20 %	0.53 (0.21-1.34)
ADC change: Reduced vs Not reduced (resected after NAC)α	7 vs 15	100 %	vs	34 %	**0.21 (0.00-)§**	57 %	vs	31 %	0.52 (0.12-1.89)
ADC change: Reduced vs Not reduced (all NAC+targeted)α	9 vs 15	57 %	vs	27 %	0.37 (0.10-1.36)	44 %	vs	27 %	0.68 (0.25-1.84)
ADC change: Reduced vs Not reduced (all resected after NAC+targeted)α	6 vs 13	100 %	vs	31 %	**0.23 (0.00-)§**	50 %	vs	31 %	0.58 (0.16-2.15)
ADC change in peripheryβ: Reduced vs Not reduced (all NAC)α	10 vs 11	23 %	vs	38 %	1.10 (0.34-3.63)	27 %	vs	36 %	1.15 (0.42-3.22)
ADC change in peripheryβ: Reduced vs Not reduced (resected)α	7 vs 7	43 %	vs	54 %	1.02 (1.69-6.23)	21 %	vs	57 %	1.60 (3.37-6.64)
MRI RECIST criteria: Partial response vs Stable disease (all NAC)	20 vs 19	62 %	vs	26 %	0.69 (0.27-1.76)	46 %	vs	25 %	0.58 (0.26-1.30)
Morphological MRI criteria: Optimal or partial response vs No response (all NAC)	22 vs 16	47 %	vs	20 %	0.44 (0.18-1.01)	40 %	vs	22 %	0.81 (0.37-1.79)
Tumour vitality: 0-30% vs 31-100%	21 vs 14	39 %	vs	68 %	2.23 (0.70-7.54)	26 %	vs	45 %	1.69 (0.69-4.15)
TRG 1-3 vs 4-5	26 vs 9	45 %	vs	67 %	1.71 (0.47-6.28)	35 %	vs	28 %	0.90 (0.35-2.32)
mTRG 1-3 vs 4-5	28 vs 7	52 %	vs	50 %	0.64 (0.14-2.91)	38 %	vs	19 %	0.77 (0.28-2.12)

* HR = Hazard ratio, CI = Confidence interval. § HR cannot be accurately calculated because there are zero events in the group "ADC reduced". α "reduced" used for ADC values that decreased during systemic therapy and "not reduced" for ADC values which either increased or did not change. β ADC change in periphery refers to the peripheral lowest ADC part of the tumour.

Supplementary table 5. Association between apparent diffusion coefficient at peripheral lowest ADC area (ADC periphery) at baseline, at response evaluation and change in ADC with neoadjuvant or conversion chemotherapy (NAC) versus RECIST and morphologic response criteria, tumour vitality, tumour regression grade (TRG) and modified tumour regression grade (mTRG).

		All NAC	Baseline ADC periphery±			Response ADC periphery±			Change in ADC periphery± with NAC		
			ADC<0.92	ADC≥0.92	RR (95% CI)**	ADC<1.13	ADC≥1.13	RR (95% CI) §	Reduced	Not reduced	RR (95% CI) ¥
**Radiology after chemotherapy**		**26**	**13**	**13**		**12**	**12**		**10**	**11**	
MRI RECIST criteria	Partial response	11 (42%)	6 (46%)	5 (38%)		8 (67%)	2 (17%)		6 (60%)	3 (27%)	
	Stable disease	15 (58%)	7 (54%)	8 (62%)	0.88 (0.42-1.74)	4 (33%)	10 (83%)	**0.40 (0.17-0.93)**	4 (40%)	8 (73%)	0.55 (0.24-1.21)
Morphological MRI criteria*	Optimal response	8 (31%)	6 (46%)	2 (15%)		4 (33%)	2 (17%)		2 (20%)	3 (27%)	
	Partial response	8 (31%)	4 (31%)	4 (31%)		5 (42%)	3 (25%)		5 (50%)	2 (18%)	
	No response	10 (38%)	3 (23%)	7 (54%)	0.43 (0.14-1.26)	3 (25%)	7 (58%)	0.43 (0.14-1.22)	3 (30%)	6 (55%)	0.55 (0.18-1.56)
**Histology after chemotherapy**		**22**	**13**	**9**		**10**	**6**		**7**	**7**	
Tumour vitality (%)	0	3 (14%)	1 (8%)	2 (22%)		2 (20%)	0 (0%)		2 (29%)	0 (0%)	
	1-30	11 (50%)	6 (46%)	5 (56%)		4 (40%)	3 (50%)		2 (29%)	4 (57%)	
	31-100	8 (36%)	6 (46%)	2 (22%)	2.08 (0.61-8.79)α	4 (40%)	3 (50%)	0.80 (0.28-2.51)α	3 (43%)	3 (43%)	1.00 (0.30-3.35)α
TRG	1-2	10 (45%)	5 (38%)	5 (56%)		4 (40%)	1 (17%)		3 (43%)	2 (29%)	
	3	7 (32%)	4 (31%)	3 (33%)		3 (30%)	5 (83%)		1 (14%)	5 (71%)	
	4-5	5 (23%)	4 (31%)	1 (11%)	2.77 (0.53-31.2)β	3 (30%)	0 (0%)	3.90 (0.50-38.8)	3 (43%)	0 (0%)	6.43 (0.78-72.5)
mTRG	1-2	10 (45%)	5 (38%)	5 (56%)		5 (50%)	1 (17%)		3 (43%)	2 (29%)	
	3	8 (36%)	5 (38%)	3 (33%)		2 (20%)	5 (83%)		1 (14%)	5 (71%)	
	4-5	4 (18%)	3 (23%)	1 (11%)	2.08 (0.34-24.40) β	3 (30%)	0 (0%)	3.90 (0.27-16.3) β	3 (43%)	0 (0%)	6.43 (0.78-72.5) β

± The peripheral lowest ADC part of the tumour. * MRI was not diagnostic for morphology because of motion artefact N=1. ** RR = Occurrence of outcome if ADC<0.92 / Occurrence of outcome if ADC≥0.92. CI = Confidence interval. § RR = Occurrence of outcome if ADC<1.13 / Occurrence of outcome if ADC≥1.13. ¥ RR = Occurrence of outcome if ADC reduced i.e. decreasing with NAC / Occurrence of outcome if ADC not reduced with NAC. α Comparison groups for RR vitality 0-30% vs. 31-100%. β Comparison groups for RR TRG and mTRG 1-3 vs. 4-5.

Supplementary table 6. Association between MRI RECIST 1.1 criteria and morphological MRI response criteria versus tumour vitality, tumour regression grade (TRG) and modified tumour regression grade (mTRG).

**Radiology after chemotherapy**		**All NAC**	**MRI RECIST criteria**			**Morphological MRI criteria**			
			Partial response (PR)	Stable disease (SD)	RR (95% CI)*	Optimal response	Partial response	No response	RR (95% CI)**
		**35**	**17**	**10**		**11**	**9**	**7**	
Tumour vitality (%)	0	5 (17%)	5 (29%)	0 (0%)		2 (18%)	2 (22%)	1 (14%)	
	1-30	16 (53%)	8 (47%)	6 (60%)		5 (46%)	6 (67%)	3 (43%)	
	31-100	9 (30%)	4 (24%)	4 (40%)	0.59 (0.20-1.81)α	4 (36%)	1 (11%)	3 (43%)	0.58 (0.20-1.88)α
TRG	1-2	14 (40%)	8 (47%)	3 (30%)		5 (46%)	4 (44%)	2 (29%)	
	3	12 (34%)	6 (35%)	5 (50%)		4 (36%)	4 (44%)	3 (43%)	
	4-5	9 (26%)	3 (18%)	2 (20%)	0.88 (0.22-4.38)β	2 (18%)	1 (11%)	2 (29%)	0.53 (0.13-2.58)β
mTRG	1-2	16 (46%)	9 (53%)	4 (40%)		5 (46%)	6 (67%)	2 (29%)	
	3	12 (34%)	6 (35%)	4 (20%)		5 (46%)	2 (22%)	3 (43%)	
	4-5	7 (20%)	2 (12%)	2 (20%)	0.59 (0.10-3.51)γ	1 (9%)	1 (11%)	2 (29%)	0.35 (0.06-2.06)γ

* RR = Occurrence of outcome if PR/ Occurrence of outcome if SD. CI = Confidence interval. ** RR = Occurrence of outcome if optimal or partial response / Occurrence of outcome if no response.
Outcomes: α Tumour vitality >30%; β TRG 4-5; γ mTRG 4-5.

RAXO trial

A population-based prospective study to evaluate clinical behaviour, resectability and survival in metastatic colorectal cancer patients in Finland

Date of Protocol: 29.8.2011_FINAL
3.12.2015_Version_2.0
30.10.2016_Version_3.1
07.05.2017_Version_3.2

SUMMARY

**Protocol title**:	A population-based prospective study to evaluate clinical behaviour, resectability and survival in 1st line metastatic colorectal cancer (CRC) patients in Finland<br><br>The RAXO trial
**Protocol version:**	07.05.2017_Version_3.2
**Principal Investigator**	Helena Isoniemi; MD, PhD; professor<br>Pia Österlund; MD, PhD; associate professor
**Study Sponsor**	Academic sponsorship
**EudraCT number**	2011-003158-24
**Project phase**	1) Prospective clinical trial and<br>2) Data collection trial
**Indication**	Metastatic cancer of the colon or rectum
**Objectives**	Primary objective:<br>• To assess clinical behaviour of metastatic colorectal cancer and overall resectability, postoperative morbidity and outcomes after resection<br>Secondary objectives:<br>• To assess treatments for mCRC<br>• To assess efficacy of chemotherapy and targeted drugs with overall response rates (ORR), failure free survival (FFS), progression free survival (PFS), and overall survival (OS)<br>• To radiologically assess tumour density and morphology, and assess alternative radiologic response evaluation in comparison with RECIST response criteria<br>• To evaluate whole blood, plasma, serum and tumour block biomarkers and DNA polymorphisms that may predict drug effects, resectability and clinical behaviour of the tumour in the prospective cohort.<br>• Biomarker evaluation from diagnostic samples, mainly tumour blocks, in the retrospective data collection trial in order to verify diagnosis and predictive & prognostic markers (from prospective clinical trial) in a big population based series.<br>• Quality of life and health related qualify of life, Cost-utility, QALY and cost-benefit assessment in the subpopulation of 100-200 patients.
**Planned sample size**	In total 1000 patients will be recruited in the clinical trial part of the study. Recruitment to the clinical trial will be between November 2011 and December 2018. Population based

	assessment in the data collection trial during the time period of recruitment to the clinical trial.
**No. of centres**	All hospitals treating colorectal cancer in Finland (appr. 20)
**Selection criteria**	**Inclusion criteria**<br>1. Patients with histologically confirmed CRC, who are scheduled to start or are getting first line chemotherapy for metastatic disease<br>2. Age ≥ 18<br>3. Metastatic disease (including locally advanced disease not amenable with surgery and/or (chemo)radiotherapy)<br>4. Signed written informed consent according to ICH/GCP and the local regulations (approved by the Independent Ethics Committee [IEC]) will be obtained prior to study<br>5. No informed consent will be obtained from patients participating in the data collection study obtaining data from hospital charts. No blood sampling, nor contacting of patients will be performed.
**Resectability**	Patients will be centrally assessed for resectability at baseline and thereafter for a maximum of 3 times (mainly for liver and lung metastasectomies) every 8-12 weeks.
**Standard medical treatment**	In each clinic standard medical treatment will be administered until disease progression, unless unacceptable drugtoxicity is experienced or until resectability is achieved. At least 3 months of the same chemotherapy treatment should be given at the physicians discretion following metastasectomy.
**Study procedures**	**Informed consent:**<br>Separate written informed consent must be obtained before the patient participates in the prospective resectability evaluation study or in the blood sampling for biomarkers.
	**Screening:**<br>Baseline screening includes following assessments:<br>• According to local standard practice:<br>- Demographic data, medical history, cancer/treatment history and concomitant medications<br>- ECOG performance status and physical examination<br>- Tumour assessments i.e. whole body CT (in combination with MRI and PET according to subprotocols or local standards) before treatment starts<br>- Central assessment of resectability initiated<br>- Whole blood for DNA , plasma and serum at the baseline<br>- Tumour blocks collected

	<u>The following data will be collected during treatment:</u><br>• According to local standard practice:<br>- Spontaneous adverse event reporting according to local regulations, without data collection for the study purposes<br>- Monitoring of concomitant diseases, treatments, medications and compliance to drugs<br>- Physical examination<br>- Assessments of tumour response using whole-body CT (or MRI/PET according to subprotocols)<br>- Confirmation of overall response<br>• Resectability will be assessed centrally after baseline for a maximum of 3 times every 8-12 weeks during the treatment<br>• Plasma and serum will be collected at the same time as tumour assessment and after last chemotherapy cycle
	<u>The following data will be collected during follow-up:</u><br>• According to local standard practice:<br>- Assessments of tumour response<br>- Subsequent treatments<br>- Spontaneous adverse event reporting, without data collection for the study purposes
**Data collection procedures**	In addition to the 1000 consenting patients participating in the clinical study; the data collection cohort will gather population based information from the cancer registry and from hospital charts for metastatic colorectal cancer patients. Similar inclusion criteria will be used, but no blood sampling or patient contacting will be performed. Inclusion period will be identical with the clinical study.
**Statistical considerations**	The primary objective is to assess clinical behaviour of mCRC and the overall resectability rate, postoperative morbidity and outcomes after resection, compared between chemotherapy regimens. The secondary objectives, i.e. treatments, RR, FFS, PFS, and OS will also be assessed overall and compared between chemotherapy regimens.<br><br>The planned size of 1000 patients (the expected Finnish yearly patient population) is considered to provide very sufficient precision for the clinical behaviour of mCRC and for assessment of overall resectability rate. Based on historical data, among 20% (200) of the Finnish yearly population are expected to have liver only disease with a resectability rate of 20% (40). With this sample size the width of the 95% confidence interval of the resectability rate will be approximately ± 5.5%.

**Analysis plan**	The primary analyses will be based on the intent-to treat population, which will include all eligible patients.<br><br>A 95 % confidence interval will be calculated for the overall resectability rate. Comparisons between chemotherapy regimens will be done using a chi-square test as well as with a logistic regression model will be used, if feasible. Resection outcome and postoperative morbidity will be presented at least by descriptive statistics.<br><br>Survival outcomes, i.e. FFS, PFS, and OS will be estimated for the overall population and for all chemotherapy regimens using the Kaplan-Meier approach. In addition, these parameters will be analyzed using the Cox-proportional hazard model.<br><br>The RR will be analyzed using chi-square test.<br><br>Radiological assessments with tumour density and morphology will be analyzed using analysis of covariance ANCOVA, with the RECIST response criteria as a covariate.
**Duration of the study**	It is expected that the first patient is enrolled in 2011 and the enrolment will be completed during 2018. Patients will be followed-up for 10 years after inclusion, until 2025.

STUDY RELATED ASSESSMENTS

Schedule	Screening / Baseline	Treatment[1] Every 8-12 weeks	Post-treatment follow up
Eligibility assessment	X		
Informed consent	X		
Demographic data	X[1]		
Medical & cancer treatment history. Concomitant medications.	X[1]	X[1]	X[1]
Tumour assessment (preferably whole body CT)	X	X[2][3]	X[4]
Central assessment of resectability	X	X[3]	
ECOG performance status	X[1]	X[1]	X[1]
Physical examination	X[1]	X[1]	X[1]
Research blood samples[5]	X	X	X
Follow up on disease progression, anticancer therapies and survival			X

(1) According to local standard practice
(2) Response verification recommended after 4-9 weeks
(3) Central assessment of resectability will be performed every 8-12 weeks for a maximum of three times
(4) If no progressive disease seen when coming off study
(5)Whole blood (10 ml EDTA-tube) for DNA at the baseline. Plasma and serum, in 30+20ml EDTA/heparin/citrate-tube and 20ml serum/gel/vacutainer-tube), will be collected at the baseline and thereafter every 8-12 weeks (at the same time as tumour assessment) and after last chemotherapy cycle

TABLE OF CONTENT

1. BACKGROUND 9

1.1. Disease Background 9

1.2. Chemotherapeutic agents for Colorectal Cancer 9
1.2.1. Combination chemotherapy 9

1.3. Biological therapies 1st line Colorectal Cancer 10

1.4. Resection of Liver Metastasis 10

1.5. Resection of lung metastases 11

1.6. Assessment of resectabilityy of liver and lung metastases 12

1.7. Radiology assessment 12

1.8. Rationale for health-related quality of life and biomarker assessments 12

2. STUDY OBJECTIVES 13

2.1. Primary objectives 13

2.2. Secondary objectives 13

3. STUDY DURATION 14

4. SELECTION CRITERIA 14

4.1. Inclusion Criteria 14

5. STUDY DESIGN 14

5.1. Design 14

5.2. Study procedures per visit 14
5.2.1. Screening and Baseline 14
5.2.2. Treatment Phase 15
5.2.3. Post-Treatment Follow Up 15
5.2.4. Biological samples 15
5.2.5. Termination of Study 16

6. STUDY MEDICATION 16

6.1. Standard medical treatment for mcrc 16
6.1.1. Standard medical treatment in connection with surgery 16

6.2. Treatment Duration 16

6.3. Concomitant Treatment & Therapy 16
6.3.1. Other Medication 16

6.3.2. Radiation therapy 16

7. PREMATURE WITHDRAWAL 17

7.1. Withdrawal From Standard Medical Treatment 17

8. WARNINGS AND PRECAUTIONS OF MEDICAL TREATMENT 17

9. SAFETY PARAMETERS 17

10. SERIOUS ADVERSE EVENT REPORTING 17

11. STATISTICAL CONSIDERATIONS 18

11.1. Sample size calculation 18

11.2. Analysis Population 18

11.3. Analysis Plan 18

12. GLOSSARY AND DEFINITION OF TERMS USED 20

13. REFERENCES 21

1. BACKGROUND

1.1. Disease Background

Colorectal cancer is one of the most frequent malignancies, second to breast cancer in women and third to prostate and lung cancer in men [1,2]. The prognosis for the individual patient is dependent upon the extent of the disease. The 5-year survival rate is over 60% in individuals with resectable cancer, but less than 5% in individuals with distant metastatic disease [2-5].

The clinical course of mCRC is of special interest. Sites of metastases primarily is of crucial value whether they are or going to be operable (typically lung or liver metastases) or not (eg. bone metastases). Ten per cent of patients with metastases confined to the liver are considered initially resectable and another 10-15% may be rendered resectable with efficient treatment, but the corresponding figures for lung metastasectomies are unknown. To the best of our knowledge this has not been assessed population based with central resectability evaluation. Co-morbidities and patient/caregiver preference in choice of active treatment and best supportive care has not been thoroughly evaluated in population based manner in mCRC.

1.2. Chemotherapeutic agents for Colorectal Cancer

Most metastatic colorectal cancer patients are treated with chemotherapy with palliative intent. In locally advanced and distant metastatic disease 5-fluorouracil (5-FU) based chemotherapy improves quality of life and survival compared with best supportive care alone [2,3,6]. Intravenous 5-FU is most efficient when combined with folinic acid (leucovorin, LV) and may be dosed as repeated bolus injections or short/long infusions [7]. Capecitabine (Xeloda™) is an oral fluoropyrimidine carbamate rationally designed to generate 5-FU preferentially in tumour tissue through exploitation of high intratumoural concentrations of thymidine phosphorylase [8].

Oxaliplatin is a platinum derivative in which the platinum atom is complexed with a 1,2 diaminocyclohexane (DACH) and with an oxalate ligand as a leaving group [9]. Oxaliplatin has been widely studied and is active as monotherapy in front-line or subsequent therapy settings in patients with mCRC [10-13].

Irinotecan is a semi-synthetic derivative of camptothecin and belong to the class of topoisomerase I inhibitors. The efficacy of irinotecan given as a single agent has been assessed in first and second line settings [14, 15].

1.2.1. Combination chemotherapy

Phase III trial results showed in 2000 the superiority of irinotecan or oxaliplatin as first-line treatment in combination with 5-FU/LV, compared to 5-FU/LV therapy alone [16-18]. Thus combination chemotherapy became the standard of care and new combination chemotherapy regimens have been explored.

Irinotecan in combination with both bolus and infusional 5-FU/LV has been shown to improve efficacy compared with 5-FU/LV alone in MCRC [8-9]. Also the combination of capecitabine plus irinotecan every 3 weeks (XELIRI regimen) has shown feasible safety and comparable efficacy [19, 20].

Oxaliplatin in combination with bolus and infusional 5-FU/LV has shown improved response rates and progression free as well as overall survival [10]. Oxaliplatin combinations has thus subsequently been evaluated also in combination with chronomodulated 5-FU and oral prodrugs. Capecitabine combinations (XELOX) has shown comparable efficacy and tolerability as infusional 5-FU based chemotherapy [19-22].

The triple combination of irinotecan, oxaliplatin and 5-fluoruracil has been evaluated in 2 randomized studies. In the Falcone study, the triple combination had superior efficacy compared with the FOLFIRI arm. Interestingly a high resection rate was seen in the Falcone study; R0 secondary resection rate was 15 vs. 6% in total population and 36 vs. 12% in the liver only metastases population [23]. In the Souglakos study, no significant benefit for the triple combination was seen [24]. This triple combination has though been considered too toxic in routine clinical care and leaves few options for further lines of chemotherapy. Modern combinations with targeted treatments (i.e. biologics) such as bevacizumab, cetuximab and panitumumab maybe too toxic in conjunction with triple chemotherapy.

1.3. Biological therapies 1st line Colorectal Cancer

Bevacizumab (Avastin™) is a humanised monoclonal antibody targeting vascular endothelial growth factor (VEGF). VEGF-A which is a ligand with a central role in signalling pathways controlling tumour blood vessel development and survival [25-28]. Several large trials have demonstrated that bevacizumab improves the efficacy of fluoropyrimidine-based chemotherapy in previously untreated patients with metastatic CRC [29-30,22] improving significantly overall survival, progression-free survival, response rate, and response duration. In addition, safety and efficacy of first-line bevacizumab combined with various chemotherapies in patients with metastatic CRC have been demonstrated in phase IV studies [31-33].

Cetuximab (Erbitux™) and panitumumab (Vectibix™) are monoclonal antibodies against the epidermal growth factor receptor (EGFR) [34-35]. The efficacy of monoclonal antibodies against EGFR is limited to KRAS wild type tumours [36-37]. Both drugs first showed efficacy in later lines of therapy both as monotherapy [38-39] and in combination with irinotecan [40]. Recently also in first and second line settings [41-42].

Cetuximab has shown superior efficacy and resectability in first line in combination with irinotecan and 5-FU/LV (FOLFIRI) [43]. Oxaliplatin and 5-FU based combinations to cetuximab have shown conflicting results and need further evaluation [44]. The biological doublet with Cetuximab and Bevacizumab showed inferiority [45].

Panitumumab as first line therapy has shown superior efficacy and resectability in combination with oxaliplatin and 5-FU/LV (FOLFOX) [46]. The biology doublet of panitumumab and bevacizumab in first line showed inferiority especially in combination with FOLFOX and no benefit in patients with FOLFIRI [47].

1.4. Resection of Liver Metastasis

The liver is the most common site of haematogenous metastasis from gastrointestinal malignancies due to portal venous blood flow from the intestine.

In the past patients with liver metastasis were often deemed inoperable. As a result of improved techniques, major hepatic resection is nowadays performed with acceptable morbidity and low perioperative mortality, under 5% in major hepatobiliary centres [48]. For patients with mCRC liver resection is the only available treatment with an option of long-term survival and also prolonged disease-free survival. Several reports have established the efficacy of surgical resection in selected patients with 5-year survival rates ranging from 37 to 71% [49-52].

However curative operation can be performed only in a small minority of all patients with colorectal metastases confined to the liver and no survival benefit is obtained from incomplete resection [54]. Today the amount of patients with curative intent surgery can be increased after down staging initially unresectable lesions by chemotherapy [53].

Neoadjuvant chemotherapy for two to six months with regular re-evaluation for resectability has been advocated as the optimal strategy to maximize respectability [48,54]. Long oxaliplatin

exposure has been linked with blue liver causing increased bleeding in liver resections [55] and long irinotecan based chemotherapy causes steatohepatosis, which may increase morbidity in conjunction with liver resections [56]. Recently published data suggest that bevacizumab may be preventing liver injury from chemotherapy in conjunction with neoadjuvant chemotherapy [57]. Thus routine re-evaluation for liver resectability is done for a maximum of three times during first line chemotherapy.

There is currently one report of a randomized controlled trial evaluating liver resection alone with neoadjuvant and adjuvant therapy combined with liver resection, showing an advantage to the outcome of hepatic resection [53]. Some recent reports and reviews propose an advantage using adjuvant chemotherapy after liver resection [48,54,58,59]. With the development of better chemotherapeutic agents, which may eradicate residual microscopic tumour cell deposits in the liver and elsewhere, partial hepatectomy to remove focal macroscopically observed metastases is likely to be more common and more effective.

1.5. Resection of lung metastases

Lung is the second most common site of metastases of CRC. CRC is recognized to be the most common primary histology for patients with potentially resectable pulmonary metastases. However the incidence of isolated lung metastases without associated metastases for CRC patients is low (<10%).

No randomized controlled trials exist to date to analyze the outcomes of patients who underwent resection of pulmonary metastases secondary to colorectal cancer. However, several centres have published results from case series, showing 5-year survival of 29-56% and a median survival from 47-74 months [60-63].

Several clinical patient series describe criteria for pulmonary metastasectomy. However, no prospective randomized trials for pulmonary metastasectomy makes it difficult to summarize and evaluate the effectiveness of this operation

There has been data reported that indicates that patients with history of previous liver metastases have a higher risk of tumour recurrence and a decreased survival in comparison with patients who underwent surgery for lung-only CRC metastases [64]. On the other hand, there are also reports concluding that resectable or resected liver metastases might not impact the survival after pulmonary metastasectomy [65].

Different reviews of studies analyzing prognostic factors differ. Thus, there are reviews reporting that number and location of metastases, disease-free interval between resection primary colorectal cancer and detection of pulmonary metastases, pre-resection CEA level, thoracic lymph-node metastasis, level of prethoracotomy serum-carcinoembryonic antigen, age, gender, site and stage are prognostic factors for improved survival rates [65-66].

Still, most reviews of studies analyzing prognostic factors such as number of nodules, size of the dominant nodule, disease-free interval, or use of chemotherapy to mention just a few, found no prognostic significance [66-67].

Surgical resection is the primary treatment modality for pulmonary metastases in colorectal cancers in patients who meet the criteria for potentially curative operation. United Kingdom National Institute for Clinical Excellence guidance makes a similar recommendation. The National Comprehensive Cancer Network Clinical Practice Guidelines in Oncology precondition for potentially curative operation as follows:

- the metastases seem to be technically resectable,
- the general and functional risks are tolerable,
- the primary tumour is controlled, and
- no extrathoracic lesions are detected (with the exception of hepatic lesions in which it is possible to completely remove both hepatic and pulmonary metastases).

The role of chemotherapy in the treatment of patients with metastatic colorectal cancer is evolving. Although in most studies chemotherapy has been a standard therapy for metastatic colorectal cancer, the impact of neoadjuvant and adjuvant chemotherapy in the context with pulmonary metastasectomy on long-term survival had not been sufficiently addressed [66].

The integration of systemic targeted therapy should be considered in decision making regarding candidacy and timing for surgery.

1.6. ASSESSMENT OF RESECTABILITYY OF LIVER AND LUNG METASTASES

Patients will be centrally assessed for resectability at baseline and after 8-12 weeks during chemotherapy maximally three times. Treatment with bevacizumab should be stopped at least 4 to 6 weeks before surgical intervention in patients who are to be referred for resection. During this time the patient may receive one or more chemotherapy doses, as clinically appropriate. Chemotherapy and biologicals may be restarted 4 weeks after surgery and bevacizumab when wound healing is complete

1.7. RADIOLOGY ASSESSMENT

Radiological assessment with whole-body CT two-monthly has been standard. WHO criteria for response were defined in 1982 and replaced by RECIST criteria in 2000. These RECIST criteria have been revised recently [68] but still leaves open the question of how to evaluate the efficacy of chemotherapy, especially in combination with biologicals, radiologically. In GIST for example metabolic activity assessed with PET was found much more reliable [69-74]. So far PET has not shown significant advantage over whole-body CT in response evaluation [75] and the role of PET needs to be established.

MRI of liver is another question not fully answered. Over the past decade liver MRI has been recommended when liver resection has been evaluated but the development of the CT technology has made the role of MRI debatable. Recently reports of altered density and morphology of for example liver metastases after chemotherapy and biologicals has been seen on radiology [76].

This study aims to explore improved radiological evaluation methods in mCRC compared with RECIST CTs.

1.8. RATIONALE FOR HEALTH-RELATED QUALITY OF LIFE AND BIOMARKER ASSESSMENTS

The study will be performed in order to analyse the clinical behaviour and treatments for mCRC population based. Central assessment of resectability will also provide a more reliable estimate of the respectability rate in Finnish metastatic CRC patients. The rationale for this study is to evaluate which treatment modalities result in higher resectability rates as well as a longer progression free survival and overall survival. Improved radiologic assessment methods and prognostic factors will also be evaluated.

Health related quality of life (HR QoL) issues and cost-utility, cost-benefit and quality adjusted life year gain (QALY) is becoming increasingly important with prolonged survival in this patient population due to improving treatment alternatives. Therefore QoL questionnaires EORTC QLQ-C30 and CR29, EQ5 and 15D will be administered to new patients to be included in the prospective clinical study, estimated 150-200 patients, which is a fair subgroup for these analysis.

Biomarkers and personalized medicine is becoming increasingly important in treatment of metastatic colorectal cancer. Prognostic markers as BRAF and predictive markers as KRAS and NRAS are used in clinical practise but several other markers are to be validated.

The inclusion criteria for the RAXO study are patients with first line treatment and therefore a significant proportion of metastatic patients with aggressive disease, old age and comorbidities are not consented and thus participate in the data collection trial. Many discrepancies for example in BRAF mutation frequency have been noted among clinical study patients with a frequency of 4 to 8% and over 20% in more population-based series (as the data collection trial) (Sörbye et al, 2015, Annika Ålgars personal communication 7.9.2016). Systemic consenting of patients to the prospective clinical study was not performed in the early days of the inclusion period due to logistic reasons.

The prospective trial will consist of slightly selective patients and therefore the diagnostic samples of the data collection trial identified via the cancer registry, the hospital and National institute of health and welfare (THL) registries are used to verify diagnosis and predictive and prognostic markers population based.

Samples up til 1st September 2013 have been transferred or are due to be transferred to the Biobanks, they are used with permission by the Biobanks. Samples from 1st September 2013 are under the biobank laws of Finland but very few patients have been given the opportunity to consent especially during the first years. Therefore they cannot be used under this permission and due to the nature of metastatic disease the majority of patients cannot be consented as of now. The majority of cases before the biobank era are also deceased and cannot consent. Cases that have declined participation into the RAXO-study will not be used in the biomarker population.

The diagnostic samples, mainly tumour tissue from data collection trial, are clinically relevant when they provide population-based information about the nature of the disease and treatment options with valuable prognostic and predictive markers benefitting metastatic colorectal patients in the future. Permission for use of these diagnostic samples are requested from VALVIRA and the ethical board.

2. STUDY OBJECTIVES

2.1. PRIMARY OBJECTIVES

- To assess clinical behaviour of metastatic colorectal cancer and overall resectability, postoperative morbidity and outcomes after resection

2.2. SECONDARY OBJECTIVES

- To assess treatments for mCRC
- To assess efficacy of chemotherapy and targeted drugs wtih overall response rates (ORR), failure free survival (FFS), progression free survival (PFS) and overall survival (OS)
- To radiologically assess tumour density and morphology, and assess alternative radiologic response evaluation in comparison with RECIST response criteria
- To evalute whole blood, plasma, serum and tumour block biomarkers and DNA polymorphisms that may predict drug effects, resectability and clinical behaviour of the tumour
- Biomarker evaluation from diagnostic samples, mainly tumour blocks, in the data collection trial in order to verify diagnosis and predictive & prognostic markers (from prospective clinical cohort) in a big population based series.
- Quality of life and health related qualify of life, Cost-utility, QALY and cost-benefit assessment in the subpopulation of 100-200 patients.

3. STUDY DURATION

It is expected that the first patient is enrolled in 2011 and the enrolment will be completed during 2018. Patients will be followed-up for 10 years after inclusion, until 2025.

4. SELECTION CRITERIA

4.1. INCLUSION CRITERIA

1. Patients with histologically confirmed CRC, who are scheduled to start or are getting first line chemotherapy for metastatic disease
2. Age ≥ 18
3. Metastatic disease (including locally advanced disease not amenable with surgery and/or (chemo)radiotherapy)
4. Signed written informed consent according to ICH/GCP and the local regulations (approved by the Independent Ethics Committee) will be obtained prior to study
5. No informed consent will be obtained from patients participating in the data collection study obtaining data from hospital charts without blood sampling and contacting of patients

5. STUDY DESIGN

5.1. DESIGN

1) Prospective clinical trial and 2) data collection trial.

5.2. STUDY PROCEDURES PER VISIT

Please refer to the Assessment flow chart (Table 1) for an overview.

5.2.1. SCREENING AND BASELINE

Signed informed consent has to be obtained from all patients prior to blood sampling and central assessment of resectability. The investigator will register the consenting patient at the co-ordinating centre. The following data will be collected and recorded:

<u>Baseline/screening includes following assessments:</u>

- According to local standard practice:
- Demographic data, medical history, cancer/treatment history and concomitant medications
- ECOG performance status and physical examination
- Tumour assessments i.e. whole body CT (in combination with MRI and PET according to subprotocols or local standards) before treatment starts
- Central assessment of resectability initiated
- Whole blood for DNA, plasma and serum at the baseline
- Tumour blocks collected

5.2.2. Treatment Phase

Patients will receive the local standard of care treatment and according to standard practice they will have scheduled visits in conjunction with treatment infusions.

<u>The following data will be collected during treatment:</u>

- According to local standard practice:
- Spontaneous adverse event reporting according to local regulations, without data collection for the study purposes
- Monitoring of concomitant diseases, treatments, medications and compliance to drugs
- Physical examination
- Assessments of tumour response using whole-body CT (or MRI/PET according to subprotocols)
- Confirmation of overall response
- Resectability will be assessed centrally after baseline for a maximum of 3 times every 8-12 weeks during the treatment
- Plasma and serum will be collected at the same time as tumour assessment and after the last chemotherapy cycle

5.2.3. Post-Treatment Follow Up

First line cancer treatment will be terminated when disease progression is identified or if toxicity is experienced that in the view of the patient or doctor is unacceptable.

<u>The following data will be collected during follow-up:</u>

- According to local standard practice:
- Assessments of tumour response
- Subsequent treatments
- Spontaneous adverse event reporting, without data collection for the study purposes

5.2.4. Biological samples

Blood samples for biomarker analyses will be collected from those patients who give the consent for the biomarker study. Blood samples will be collected at the baseline, at efficacy assessments during the treatment phase and at the final visit.

Sample collection will include whole blood (10 ml EDTA-tube) for DNA at the baseline. Plasma and serum, 18ml and 12 ml each, (in 30ml EDTA/heparin/citrate-tube and 20ml serum/gel/vacutainer-tube), will be collected at the baseline and thereafter every 8-12 weeks (at the same time as tumour assessment) and after last chemotherapy cycle

Serum and plasma samples are collected for protein analyses (blood collected into an tube as defined in Table 1) and blood samples for DNA extraction in order to measure DNA polymorphism. The set of plasma biomarkers will be analyzed including but not limited to: biomarkers that predict the clinical behaviour of the tumour, angiogenesis, resectability and regeneration biomarkers.

Baseline sample:
- both plasma and serum samples and blood samples for DNA extraction will be collected as defined in Table 1.

Every 8-12 weeks during the treatment phase in conjunction with efficacy assessment:
- serum and plasma samples for protein analyses will be collected as defined in Table 1.

The last visit:
- serum and plasma samples for protein analyses will be collected as defined in Table 1.

5.2.5. Termination of Study

The survival status of each patient and subsequent treatments should be assessed at the end of 1st line chemotherapy, at three months intervals according to the standard visit schedule, and at the end of follow-up i.e. death of the patient, withdrawal of consent or termination of the study.

6. STUDY MEDICATION

6.1. Standard medical treatment for MCRC

The clinic's standard medical treatment will be administered until disease progression, unless unacceptable toxicity is experienced or until resectability is achieved. Stop and go strategy is used according to local standard. Progression on first line treatment is defined as when resistance to reintroduced drugs is verified.

6.1.1. Standard medical treatment in connection with surgery

Patients who are to be referred for liver or lung resection will receive scheduled treatment up to 3 weeks before surgery. No bevacizumab should be given within 4-8 weeks before (thus bevacizumab is often omitted from the last cycle) and 4 weeks after surgery. Following metastasectomy at least 3 months of the same chemotherapy treatment should be given at the physicians discretion.

6.2. Treatment Duration

Standard medical cancer treatment will be administered until disease progression is identified or if toxicity is experienced that in the view of the patient or doctor is unacceptable.

6.3. Concomitant Treatment & Therapy

6.3.1. Other Medication

Any concomitant therapy is at the clinician's discretion and should be recorded.

6.3.2. Radiation therapy

Should the patient need to undergo radiation therapy the procedure will be recorded.

7. PREMATURE WITHDRAWAL

7.1. Withdrawal From Standard Medical Treatment

All patients are allowed to withdraw from the study at any time and for whatever reason without affecting their right to an appropriate follow-up treatment.

8.WARNINGS AND PRECAUTIONS OF MEDICAL TREATMENT

For warnings and precautions concerning any of the standard medical treatments for colorectal cancer the reference document is the Summary of Product Characteristics.

9. SAFETY PARAMETERS

Safety variables according to local clinical practice will be monitored according to local standards but will not be collected for study purposes:

- Adverse events (inclusive serious adverse events)
- Vital signs
- Laboratory tests

10. SERIOUS ADVERSE EVENT REPORTING

The SAE reporting and annual safety reporting is the responsibility of the sponsor (=investigators) and are done according to the local legislation.

Progression of underlying malignancy is not reported as an adverse event if it is clearly consistent with the suspected progression of the underlying cancer as defined by RECIST criteria.

1) SUSARs

The sponsor submits SUSARs to Health authority (FIMEA).

2) SAEs

Any protocol defined Serious Adverse Events and pregnancy reports that occur during the course of the study or within 4 weeks following treatment discontinuation or completion will be collected by the sponsor periodically.

Excluded from the requirement of expedited reporting are all expected events, although serious (Grade 3 or 4), that are more common as 1% according to SPCs (summary of product characteristics) adverse event chapter. SAEs excluded from the requirement of expedited reporting are collected to AE-pages in case report forms and summarized at the end of the study.

3) AEs

AEs are collected to AE-pages in case report forms and information of these non-serious adverse events is summarized at the end of study.

The definition and reporting requirements of ICH Guideline for Clinical Safety Data Management, Definitions and Standards for Expedited Reporting, Topic E2 and local legislation will be adhered to. Complete information can be found in www.ich.org and www.nam.fi.

11. STATISTICAL CONSIDERATIONS

11.1. SAMPLE SIZE CALCULATION

The planned size of 1000 patients (the expected Finnish yearly patient population) is considered to provide very sufficient precision for the clinical behaviour of mCRC and for assessment of overall resectability rate. Based on historical data, among 20% (200) of the Finnish yearly population are expected to have liver only disease with a resectability rate of 20% (40). With this sample size the width of the 95% confidence interval of the resectability rate will be approximately ± 5.5%.

11.2. ANALYSIS POPULATION

The primary analysis of resectability rate will be based on all eligible patients who had at least one central resectability evaluation performed. The Intention-to-treat (ITT) approach, i.e. all eligible patients, will be applied on other analyses in this population based study.

11.3. ANALYSIS PLAN

This is a summary of the planned statistical analyses. The statistical analysis will be described more in detail in a separate statistical analysis plan.

The primary objectives are the clinical behaviour of mCRC and the overall resectability rate, postoperative morbidity and outcomes after resection. Different chemotherapy regimens will also be compared with respect to these parameters. A 95 % confidence interval will be calculated for these parametres. Comparisons between clinical factors and chemotherapy regimens will be done using a chi-square test as well as a logistic regression model. As the expected number of resections is quite low, resection outcome and postoperative morbidity will be summarized mainly by descriptive statistics. However, a 95 % confidence intervals will be calculated for overall resection outcome and postoperative morbidity rates.

Survival outcomes, i.e. failure free survival, progression free survival (PFS), and overall survival will be estimated for the overall population and for all chemotherapy regimens using the Kaplan-Meier approach. In addition, the Cox-proportional hazard model will be used for comparison of chemotherapy regimens in the analysis of these parameters.

The response rate will be summarized overall and the differences between chemotherapy regimens will be analyzed using chi-square test.

Tumour density and morphology will be summarized and analyzed using analysis of covariance ANCOVA, with the RECIST response criteria as a covariate.

The demographic and baseline characteristics will be summarized with descriptive statistics.

SIGNATURE

I agree to perform the clinical study according to the protocol, international good clinical practice principles and regulatory authority requirements.

PRINCIPAL INVESTIGATORS:

DATE & PLACE SIGNATURE

______________________ ____

Professor Helena Isoniemi

______________________ Helsinki University Central Hospital, Dept. of Surgery

DATE & PLACE SIGNATURE

______________________ ____

Associate professor Pia Österlund

______________________ Helsinki University Central Hospital, Dept. of Oncology

Tampere University Central Hospital, Dept. of Oncology

12. GLOSSARY AND DEFINITION OF TERMS USED

AE	Adverse event
ALAT	Alanine amino transferase
ASAT	Aspartate amino transferase
CA19-9	Cancer antigen 19-9
CEA	Carcinoembryonic antigen
CNS	Central nervous system
CPT-11	Irinotecan
CRP	C-reactive protein
CT	Computer tomography
CTCAE	Common terminology criteria for adverse events
DACH	1,2-diaminocyclohexane
DPD	Dihydropyrimidine dehydrogenase
EGFR	Epidermal growth factor receptor
FOLFOX	Infusional 5-FU, leucovorin and oxaliplatin (biweekly)
FOLFIRI	Infusional 5-FU, leucovorin and irinotecan (biweekly)
5-FU	5-fluorouracil
GCP	Good Clinical Practice
Gr	Grade
HUCH	Helsinki University Central Hospital
ICH	International Committee on Harmonisation
IEC	Independent Ethics Committee
LV	Leucovorin (folinic acid)
MRI	Magnetic resonance imaging
NCI	National Cancer Institute (of the United States of America)
NSAIDs	Non-steroidal anti-inflammatory drugs
OS	Overall survival
PFS	Progression free survival
RECIST	Response evaluation criteria in solid tumors
SAE	Serious adverse event
SUSAR	Suspected unexpected serious adverse reaction
TTP	Time to tumor progression or death
ULN	Upper limit of normal
VEGF	Vascular endothelial growth factor
WHO PS	WHO or Zubrod performance status
XELOX	Capecitabine, oxaliplatin

13. REFERENCES

1. SEER Cancer Statistics Review 1973-1999. http://seer.cancer.gov/csr/1973_1999/colorect.pdf

2. Verschraegen CF, Pazdur R. Medical management of colorectal carcinomas. Tumori 1994; 80(1): 1-11.

3. Kemeny N. Current approaches to metastatic colorectal cancer. Semin Oncol 1994; 21(4 suppl 7): 67-75.

4. Wolmark N, Rockette H, Mamounas E, et al. Clinical trial to assess the relative efficacy of fluorouracil and leucovorin, fluorouracil and levamisole, and fluorouracil, leucovorin, and levamisole in patients with Dukes' B and C carcinoma of the colon: results from National Surgical Adjuvant Breast and Bowel Project C-04. J Clin Oncol 1999, 17(11): 3553-3559.

5. Porschen R, Bermann A, Loffler T, et al. Fluorouracil plus leucovorin as effective adjuvant chemotherapy in curatively resected stage III colon cancer: results of the trial adjCCA-01. J Clin Oncol 2001, 19(6): 1787-1794.

6. Graf W, Pahlman L, Bergstrom R, Glimelius B. The relationship between an objective response to chemotherapy and survival in advanced colorectal cancer. Br J Cancer 1994; 70(3): 559-563.

7. Lokich JJ, Ahlgren JD, Gullo JJ, et al. A prospective randomized comparison of continuous infusion fluorouracil with a conventional bolus schedule in metastatic colorectal carcinoma: a Mid-Atlantic Oncology Program Study. J Clin Oncol 1989; 7: 425-432.

8. Miwa M, Ura M, Nishida M, et al. Design of a novel oral fluoropyrimidine carbamate, capecitabine, which generates 5-fluorouracil selectively in tumors by enzymes concentrated in human liver and cancer tissue. Eur J Cancer 1998; 34(8):1274-1281.

9. Sanofi-Synthelabo. Eloxatin: Summary of product characteristics.: France; August 2001.

10. Raymond E, Chaney SG, Taama A, et al. Oxaliplatin a review of preclinical and clinical studies. Ann Oncol 1998, 9 (10):1053-1071.

11. Machover D, Diaz-Rubio E, et al. Two consecutive phase II studies of Eloxatin (L-OHP) for treatment of patients with advanced colorectal carcinoma who were resistant to previous treatment with fluoropyrimidines. Ann Oncol 1996;7: 95-98.

12. Garufi D, Lévi F, Misset JL. Activity of Eloxatin in colonic cancer: a report of 25 demonstrative cases. Debiopharm Internal Report, October 1994.

13. Eloxatin^{TM} SPC (summary of product caharacteristics).

14. Shimada Y, Rougier P, Pitot H: Efficacy of CPT-11 (irinotecan) as a single agent in metastatic colorectal cancer. Eur J Cancer 1996;32A Suppl 3:S13-7.

15. Campto^{TM} SPC (summary of product characteristics)

16. Saltz LB, Cox JV, Blanke C, et al. Irinotecan plus fluorouracil and leucovorin for metastatic colorectal cancer. Irinotecan Study Group. N Engl J Med 2000; 343(13): 905-914.

17. Douillard JY, Cunningham D, Roth AD, et al. Irinotecan combined with fluorouracil compared with fluorouracil alone as first-line treatment for metastatic colorectal cancer: a multicentre randomized trial. Lancet 2000; 355 (9209): 1041-1047.

18. de Gramont A, Figer A, Seymour M, et al. Leucovorin and fluorouracil with or without oxaliplatin as first-line treatment in advanced colorectal cancer. J Clin Oncol 2000; 18: 2938-2947.

19. Diaz-Rubio E, TRJ Evans, J Tabernero et al. Capecitabine (Xeloda®) in combination with oxaliplatin: a phase I, dose-escalation study in patients with advanced or metastatic solid tumors. Ann Oncol 2002; 13: 558-565.

20. Zeuli M, Di Costanzo F, Sdrobolini A, et al. Capecitabine and oxaliplatin in advanced colorectal cancer: A dose-finding study. Ann Oncol 2002; 12: 1737-1741.

21. Cassidy J, Clarke S, Diaz-Rubio E et AL. Randomized phase III study of capesitabine plus oxaliplatin compared with fluorouracil/folinic acid plus oxaliplatin as first-line therapy for metastatic colorectal câncer. J Clin Oncol 2008; 26(12): 2006-2012.

22. Saltz Et al: Bevacizumab in combination with oxaliplatin-based chemotherapy as first line therapy in metastatic colorectal cancer: A randomized phase III study. J Clin Oncol 2008; 26(12): 2013-2019.

23. Falcone A, Ricci S, Brunetti I, Pfanner E et al.: Phase III Trial of Infusional Fluorouracil, Leucovorin, Oxaliplatin, and Irinotecan (FOLFOXIRI) Compared With Infusional Fluorouracil, Leucovorin, and Irinotecan (FOLFIRI) As First-Line Treatment for Metastatic Colorectal Cancer: The Gruppo Oncologico Nord Ovest. J Clin Oncol 2007; 25 (13): 1670-1676

24. Souglakos[1] J, Androulakis N, Syrigos K, Polyzos A et al: FOLFOXIRI (folinic acid, 5-fluorouracil, oxaliplatin and irinotecan) *vs.* FOLFIRI (folinic acid, 5-fluorouracil and irinotecan) as first-line treatment in metastatic colorectal cancer (MCC): a multicentre randomised phase III trial from the Hellenic Oncology Research Group (HORG). British Journal of Cancer 2006; 94: 798–805.

25. Achen MG, Stacker SA. The vascular endothelial growth factor family; proteins which guide the development of the vasculature. Int J Exp Pathol. 1998; 79(5):255-265.

26. Ferrara N. VEGF: an update on biological and therapeutic aspects. Curr Opin Biotechnol 2000;11:617–624.

27. Ferrara N. Role of vascular endothelial growth factor in regulation of physiological angiogenesis. Am J Physiol Cell Physiol. 2001; 280(6):C1358-1366.

28. Shibuya M. Structure and function of VEGF/VEGF-receptor system involved in angiogenesis. Cell Structure Function 2001;26:25–35.

29. Hurwitz H, Fehrenbacher L, Novotny et al. Bevacizumab plus irinotecan, fluorouracil, and leucovorin for metastatic colorectal cancer. N Engl J Med. 2004; 350(23):2335-42

30. Kabbinavar FF, Schulz J, McCleod M, et al; Addition of Bevacizumab to Bolus Fluorouracil and Leucovorin in First-Line Metastatic Colorectal Cancer: Results of a Randomized Phase II Trial. J Clin Oncol 2005; 23:3697-3705.

31. Sobrero et al: Phase IV study of bevacizuman in combination with infusional fluorouracil, leucovorin and irinotecan (FOLFIRI) in first-line metastatic colorectal cancer. Oncology 2009; 77:113-119.

32. Van Cutsem et al: Safety and efficacy of first-line bevacizumab with FOLFOX; XELOX, FOLFIRI and fluoropyrimidines in metastatic colorectal cancer: The BEAT study. Ann of Onc 20:1842-1847, 2009.

33. Grothey et al: Bevacizumab Beyond First Progression is associated with prolonged overall survival in metastatic colorectal cancer: Results from a large observational cohort study (BRiTE). J Clin Oncol 2009; 26(33) :5326-5334.

34. Markman B, Javier Ramos F, Capdevila J, Tabernero J: EGFR and KRAS in colorectal cancer. Adv Clin Chem. 2010;51:71-119.

35. Markman B, Capdevila J, Elez E, Tabernero J: New trends in epidermal growth factor receptor-directed monoclonal antibodies. Immunotherapy. 2009 Nov;1(6):965-82.

36. De Mattos-Arruda L, Dienstmann R, Tabernero: Development of Molecular Biomarkers in Individualized Treatment of Colorectal Cancer. J.Clin Colorectal Cancer. 2011 May 12.

37. Lin AY, Buckley NS, Lu AT, Kouzminova NB, Salpeter SR: Effect of KRAS mutational status in advanced colorectal cancer on the outcomes of anti-epidermal growth factor receptor monoclonal antibody therapy: a systematic review and meta-analysis.. Clin Colorectal Cancer. 2011 Mar 1;10(1):63-9.

38. Van Cutsem E, Peeters M, Siena S, Humblet Y, Hendlisz A, Neyns B, Canon JL, Van Laethem JL, Maurel J, Richardson G, Wolf M, Amado RG: Open-label phase III trial of panitumumab plus best supportive care compared with best supportive care alone in patients with chemotherapy-refractory metastatic colorectal cancer. J Clin Oncol. 2007 May 1;25(13):1658-64.

39. Jonker DJ, O'Callaghan CJ, Karapetis CS, Zalcberg JR et al: Cetuximab for the treatment of colorectal cancer. N Engl J Med. 2007 Nov 15;357(20):2040-8.

40. Cunningham D, Humblet Y, Siena S, Khayat D et al: Cetuximab monotherapy and cetuximab plus irinotecan in irinotecan-refractory metastatic colorectal cancer. N Engl J Med. 2004 Jul 22;351(4):337-45.

41. Sobrero AF, Maurel J, Fehrenbacher L et al: EPIC: Phase III Trial of Cetuximab Plus Irinotecan After Fluoropyrimidine and Oxaliplatin Failure in Patients With Metastatic Colorectal Cancer. J Clin Oncol 2008: 26:2311-2319

42. Seymour, et al: Addition of panitumumab to irinotecan: Results of PICCOLO, a randomized controlled trial in advanced colorectal cancer (aCRC). ASCO 2011 (abstract 3523)

43. Van Cutsem E, Kohne C-H, Hitre E et al: Cetuximab and Chemotherapy as Initial Treatment for Metastatic Colorectal Cancer. N Engl J Med 2009;360:1408-17.

44. Maughan TS, Adams RA, Smith CG, Meade AM et al: Addition of cetuximab to oxaliplatin-based first-line combination chemotherapy for treatment of advanced colorectal cancer: results of the randomised phase 3 MRC COIN trial. Lancet. 2011 Jun 18;377(9783):2103-14.

45. Tol J, Koopman M, Cats A, Rodenburg CJ, Creemers GJ et al: Chemotherapy, bevacizumab, and cetuximab in metastatic colorectal cancer. N Engl J Med. 2009 Feb 5;360(6):563-72.

46. Douillard JY, Siena S, Cassidy J, Tabernero J: Randomized, phase III trial of panitumumab with infusional fluorouracil, leucovorin, and oxaliplatin (FOLFOX4) versus FOLFOX4 alone as first-line treatment in patients with previously untreated metastatic colorectal cancer: the PRIME study. J Clin Oncol. 2010 Nov 1;28(31):4697-705.

47. Hecht JR, Mitchell E, Chidiac T, Scroggin C et al: . A randomized phase IIIB trial of chemotherapy, bevacizumab, and panitumumab compared with chemotherapy and bevacizumab alone for metastatic colorectal cancer. J Clin Oncol. 2009 Feb 10;27(5):672-80.

48. Benoist et al: The Role of Preoperative Chemotherapy in Patients with Resectable colorectal Liver Metastases. Ann Surg Oncol (2009) 16:2385-2390.

49. Morris EJ, Forman D, Thomas JD, Quirke P, Taylor EF, Fairley L, Cottier B, Poston G: Surgical management and outcomes of colorectal cancer liver metastases. Br J Surg. 2010 Jul;97(7):1110-8.

50. van der Pool AE, Lalmahomed ZS, de Wilt JH, Eggermont AM, Ijzermans JN, Verhoef C: Trends in treatment for synchronous colorectal liver metastases: differences in outcome before and after 2000. J Surg Oncol. 2010 Oct 1;102(5):413-8.

51. House MG, Ito H, Gönen M, Fong Y, Allen PJ, DeMatteo RP, Brennan MF, Blumgart LH, Jarnagin WR, D'Angelica MI: Survival after hepatic resection for metastatic colorectal cancer: trends in outcomes for 1,600 patients during two decades at a single institution. J Am Coll Surg. 2010 May;210(5):744-52, 752-5.

52. Aloia TA, Vauthey JN, Loyer EM, Ribero D, Pawlik TM, Wei SH, Curley SA, Zorzi D, Abdalla EK: Solitary colorectal liver metastasis: resection determines outcome. Arch Surg. 2006 May;141(5): 460-6; discussion 466-7.

53. Nordlinger et al: Perioperative chemotherapy with FOLFOX4 and surgery versus surgery alone for resectable liver metastases from colorectal cancer (EORTC Intergroup trial 40983): a randomized controlled trial. The Lancet 2008; 371:1007-1016.Mitry et al:Adjuvant chemotherapy After Potentially Curative Resection of Metastases From Colorectal Cancer: A Pooled Analysis of two Randomized Trials. J Clin Oncol 2008; 26(30): 4906-4911.

54. Chua et al: Systematic Review of Randomized and Nonrandomized Trials of the Clinical response and Outcomes of Neoadjuvant Systemic Chemotherapy for resectable colorectal Liver Metastases. Ann Surg Oncol 2010; 17(2): 492-501.

55. Mehta N, Ravikumar R, Coldham C, Buckels J, Hubscher S, Bramhall S, Wigmore S, Mayer A, Mirza D: Effect of preoperative chemotherapy on liver resection for colorectal liver metastases. Eur J Surg Oncol *2008*, 34(7), 782-786.

56. Vauthey J-N, Pawlik TM, Ribero D, Wu T-T et al.: Chemotherapy Regimen Predicts Steatohepatitis and an Increase in 90-Day Mortality After Surgery for Hepatic Colorectal Metastases. J Clin Oncol 2006: 24(13), 2065-2072.

57. Ellis LM, Curley SA, Grothey A: Surgical Resection After Downsizing of Colorectal Liver Metastasis in the Era of Bevacizumab. J Clin Oncol 2005; 23(22): 4853-4855.

58. Mahfud, Breitenstein et al: Impact of Preoperative Bevacizumab on Complications After resection of Colorectal Liver Metastases: Case-Matched Control study. World J Surg October 2010; 34(1): 92-100.

59. Kemeny N: The management of resectable and unresectable liver metastases from colorectal cancer. Curr Opin Oncol 2010 Jul;22(4):364-73.

60. Welter S, Jacobs J, Krbek T, et al.: Long-term survival after repeated resection of pulmonary metastases from colorectal cancer. Ann Thorac Surg 2007;84:203–10.

61. Rotolo N, De Monte L, Imperatori A, et al. Pulmonary resections of single metastases from colorectal cancer. Surg Oncol 2007;16:5141– 4.

62. Lo C, Chu C, Zhu T, et al. Pulmonary resection for metastases from colorectal cancer. Surgical Practice 2007;11:147–53.

63. Gilliams A. Minimally invasive treatment for liver and lung metastases in colorectal cancer. BMJ 2007:334:1056-1057.

64. Landes U, Robert J, Perneger T et al. R Predicting survival after pulmonary metastasectomy for colorectal cancer: previous liver metastases matter. BMC Surgery 2010, 10:17.

65. Kaifi J, Gusani NJ, Deshaies I et al. Indications and Approach to Surgical Resection of Lung Metastases. Journal of Surgical Oncology 2010;102:187–195.

66. Pfannschmidt J, Hoffmann H, Dienemann H. Reported Outcome Factors for Pulmonary Resection in Metastatic Colorectal Cancer. J Thorac Oncol. 2010;5: S172–S178.

67. Erhunmwunsee L, D'Amico TA. Surgical Management of Pulmonary Metastases. Ann Thorac Surg 2009;88:2052-2060.

68. Eisenhauer et al: New response evaluation criteria in solid tumors: Revised RECIST guideline (version 1.1.) Eur j Cancer, 2009; 45:228-247.

69. Choi H.:Response evaluation of gastrointestinal stromal tumors. Oncologist. 2008;13 Suppl 2:4-7. Review.

70. Benjamin RS, Choi H, Macapinlac HA, Burgess MA, Patel SR, Chen LL, Podoloff DA, Charnsangavej C.: We should desist using RECIST, at least in GIST. J Clin Oncol. 2007 May 1;25(13):1760-4.

71. Choi H, Charnsangavej C, Faria SC, Macapinlac HA, Burgess MA, Patel SR, Chen LL, Podoloff DA, Benjamin RS.: Correlation of computed tomography and positron emission tomography in patients with metastatic gastrointestinal stromal tumor treated at a single institution with imatinib mesylate: proposal of new computed tomography response criteria. J Clin Oncol. 2007 May 1;25(13):1753-9.

72. Hong X, Choi H, Loyer EM, Benjamin RS, Trent JC, Charnsangavej C.: Gastrointestinal stromal tumor: role of CT in diagnosis and in response evaluation and surveillance after treatment with imatinib. Radiographics. 2006 Mar-Apr;26(2):481-95.

73. Choi H.:Critical issues in response evaluation on computed tomography: lessons from the gastrointestinal stromal tumor model. Curr Oncol Rep. 2005 Jul;7(4):307-11.

74. Choi H, Charnsangavej C, de Castro Faria S, Tamm EP, Benjamin RS, Johnson MM, Macapinlac HA, Podoloff DA.:CT evaluation of the response of gastrointestinal stromal tumors after imatinib mesylate treatment: a quantitative analysis correlated with FDG PET findings. AJR Am J Roentgenol. 2004 Dec;183(6):1619-28.

75. Wahl et al: From RECIST to PERCIST: Evolving considerations for PET response criteria in solid tumor. J Nucl Med 50:122S-150S, 2009.

76. Chun et al: Association of Computed Tomography Morphologic Criteria With Pathologic Response and Survival in Patients Treated With Bevacizumab for Colorectal Liver Metastases. JAMA, December 2, 2009; 302(21): 2338-2344.
