# Supplementary material for: Treatment response of colorectal cancer liver metastases to neoadjuvant or conversion therapy: a prospective multicentre follow-up study using MRI, diffusion-weighted imaging and 1H-MR spectroscopy compared with histology (subgroup in the RAXO trial)
Source: ESMO Open. 2021 Jul 26;6(4):100208. doi: 10.1016/j.esmoop.2021.100208 (PMC8332656; doi:10.1016/j.esmoop.2021.100208)
Supplement: Supplementary material. Trial registration data [file mmc2.docx]

**TRIAL REGISTRATION DATA**

| **Data category** | **Information** |
| --- | --- |
| Primary registry and trial identifying number | 2011-003158-24 (EudraCT)  NCT01531621 (ClinicalTrials.gov) |
| Date of registration in primary registry | 22 September 2011 |
| Source(s) of monetary or material support | Finska Läkaresällskapet, The Finnish Cancer Foundation, Relander’s Foundation, the Competitive State Research Financing of the Expert Responsibility Area of Tampere, Helsinki and Turku, Tampere University Hospital Funds and Research Fund of Helsinki University Hospital. The infrastructure with database and study nurses were partly supported by pharmaceutical companies (Amgen—unrestricted grant, Lilly, Merck KGaA, Roche Finland, Sanofi and Servier—unrestricted grant) |
| Primary sponsor | Helsinki University Hospital |
| Contact for public queries | Pia Osterlund, pia.osterlund@helsinki.fi |
| Contact for scientific queries | Pia Osterlund  Department of Oncology, Tampere University Hospital and University of Tampere.  Teiskontie 35, 33520 Tampere, Finland  Phone +358 50 337 5000  E-mail: pia.osterlund@pshp.fi |
| Public title | MRI treatment response of colorectal cancer liver metastases |
| Scientific title | This substudy: Treatment response of colorectal cancer liver metastases to neoadjuvant or conversion therapy: a prospective multicentre follow-up study using MRI, diffusion-weighted imaging and ^1^H-MR spectroscopy compared with histology (subgroup in the RAXO trial)  Main study: A population-based prospective study to evaluate clinical behaviour, resectability and survival in 1st line metastatic colorectal cancer (CRC) patients in Finland  The RAXO trial |
| Countries of recruitment | Finland |
| Health condition(s) or problem(s) studied | Metastatic colorectal cancer |
| Intervention(s) | This substudy:  First-line neoadjuvant or conversion chemotherapy |
| Key inclusion and exclusion criteria | Ages eligible for study: over 18 years  Sexes eligible for study: all  Accepts healthy volunteers: no  Inclusion:  Patient participating in the RAXO trial in Helsinki, Oulu, Tampere or Turku University Hospital  Patients with histologically confirmed colorectal cancer with liver-only metastases, who are scheduled to start or are getting first-line chemotherapy for metastatic disease  Patient should preferably have an at least 2-cm metastasis in the right liver lobe  Signed written informed consent according to ICH/GCP and the local regulations [approved by the Independent Ethics Committee (IEC)] will be obtained before study |
| Study type | Observational multicentre phase IV cohort study  Primary purpose in this substudy: early systemic therapy response evaluation |
| Date of first enrolment | 13 February 2012 |
| Target sample size | Main study: 1000 patients  Substudy: All patient included in the main study and fulfilling the substudy inclusion criteria at participating centres |
| Final sample size | Main study: 1086 patients  Substudy: 54 patients recruited of whom 2 were excluded |
| Recruitment status | Completed |
| Primary outcomes | Substudy: To radiologically assess tumour density and morphology, and assess alternative radiologic response evaluation in comparison with RECIST response criteria and survival |
| Ethics review | Study protocol approved by Helsinki University Hospital Ethics Committee, 14 September 2011 (242/13/03/03/2011) |
| Completion date | Substudy: 4 May 2020 |
| Summary results | Main Study results first published:  14 February 2021. DOI:10.1016/j.lanepe.2021.100049  and 22 March 2021. DOI: 10.1093/bjs/znaa145  Substudy results not published before  Baseline characteristics: Table 1  Participant flow: Supplementary Figure S1, available at https://doi.org/10.1016/j.esmoop.2021.100208  Adverse events: Reporting was according to local regulations, without data collection for the study purposes  Study protocol: Main study protocol version 3.2 (7 May 2017) as supplementary file  Main findings:  Low ADC values of colorectal liver metastases before and after systemic therapy associate with better RECIST response but not with morphological MRI changes or histology.  ADC decrease with systemic therapy associates with improved overall survival. |
| IPD sharing statement | The data collected for this study can be made available to others in de-identified form after all primary and secondary endpoints have been published, in the presence of a data transfer agreement, and if the purpose of use complies with Finnish legislation. Requests for data sharing can be made to the corresponding author, including a proposal that must be approved by the steering committee. |
